# Matching action to need: an analysis of Global Burden of Disease 2017 and population health data to focus adolescent health policy and actions in Myanmar

**DOI:** 10.1080/16549716.2020.1844976

**Published:** 2021-01-15

**Authors:** Karly I. Cini, Phone Myint Win, Zay Yar Swe, Kyu Kyu Than, Thin Mar Win, Ye Win Aung, Aye Aye Myint, Nisaa R. Wulan, Lia J. Burns, Elissa C. Kennedy, Kate L. Francis, Sanda, Su Mon Myat, Sithu Swe, Aung Ko Ko, Yadanar, Margaret Hellard, Chad L. Hughes, George C. Patton, Ali H. Mokdad, Peter S. Azzopardi

**Affiliations:** a Burnet Institute, Melbourne, Australia; b Centre for Adolescent Health, Royal Children’s Hospital, Melbourne, Australia; c Murdoch Children’s Research Institute, Melbourne, Australia; d Burnet Institute Myanmar, Yangon, Myanmar; e ChildFund Vietnam, Hanoi, Vietnam; f School of Public Health and Preventive Medicine, Monash University, Melbourne, Australia; g Ministry of Health and Sports, Nay Pyi Taw, Myanmar; h World Health Organisation, Nay Pyi Taw, Myanmar; i Myanmar Youth Affair Committee, Yangon, Myanmar; j United Nations Population Fund, Yangon, Myanmar; k Department of Infectious Diseases, The Alfred Hospital, Melbourne, Australia; l, Peter Doherty Institute for Infection and Immunity, Melbourne, Australia; m School of Population and Global Health, The University of Melbourne, Melbourne, Australia; n Department of Paediatrics, The University of Melbourne, Melbourne, Australia; o Institute for Health Metrics and Evaluation, University of Washington, Seattle, WA, USA; p Wardliparingga Aboriginal Research Unit, South Australian Health and Medical Institute, University of Adelaide, Adelaide, Australia; q School of Medicine, University of Adelaide, Adelaide, Australia

**Keywords:** Adolescent health, Myanmar, morbidity, mortality, policy

## Abstract

**Background**: Myanmar is a country undergoing rapid transitions in health. Its national strategic policy for young people’s health is being revised but there is a paucity of population data to inform local priorities and needs.

**Objective**: In this paper we describe a comprehensive profile of adolescent health in Myanmar to focus policy and health actions.

**Methods**: We used available primary data, and modelled estimates from the GBD 2017, to describe health outcomes (mortality and morbidity), health risks and determinants for adolescents in Myanmar between 1990–2017. A governance group of key stakeholders guided the framing of the study, interpretation of findings, and recommendations.

**Results**: Overall health has improved for adolescents in Myanmar since 1990, however adolescent mortality remains high, particularly so for older adolescent males; all-cause mortality rate for 10–24 years was 70 per 100,000 for females and 149 per 100,000 for males (16,095 adolescent deaths in 2017). Overall, the dominant health problems were injuries for males and non-communicable disease for females in a context of ongoing burden of communicable and nutritional diseases for both sexes, and reproductive health needs for females. Health risks relating to undernutrition (thinness and anaemia) remain prevalent, with other health risks (overweight, binge alcohol use, and substance use) relatively low by global and regional standards but increasing. Gains have been made in social determinants such as adolescent fertility and modern contraception use; however, advances have been more limited in secondary education completion and engagement in employment and post education training.

**Conclusions**: These results highlight the need to focus current efforts on addressing disease and mortality experienced by adolescents in Myanmar, with a specific focus on injury, mental health and non-communicable disease.

## Background

Myanmar, a South East Asian country of 56 million and over 135 ethnic groups, has undergone rapid socio-political transition and development [1]. Between 1990 and 2017 Myanmar’s Sociodemographic Index (derived from income per capita, average educational attainment and total fertility rate) increased from 0.3 to 0.6, with its index of Health Care Access and Quality more than doubling in the same period (41.6 in 2106). Correspondingly, there have been substantial gains in health made, with life expectancy increasing 13.8 years for females and 12.4 years for males since 1990, and under-five mortality reducing from 141 per 1,000 births in 1990 to 44 per 1,000 in 2017 [2]. These transitions have brought rapid demographic shifts, with adolescents (aged 10–24 years) now representing 30% of the total population. These young people are delaying marriage and increasingly participating in secondary education [3], with the potential for driving further sustainable economic development in Myanmar. Myanmar has also seen a rapid up-take of digital media [4], with exposure to Western media and advertising likely to be influencing health behaviours and challenging social and cultural values and norms [5].

Myanmar’s Government has long recognised the need to invest in the health and wellbeing of adolescents and young people. The first National Strategic Plan (NSP) on Adolescent Health and Development was published in 2009, accompanied by the Myanmar Youth Policy in 2017 that brought a broader focus to areas outside of health [6]. The previous strategy was refreshed to the current National Strategic Plan for Young People’s Health (2016–2020) to bring a focus to seven key priority areas: sexual and reproductive health; HIV; nutrition; substance use; unintentional injuries; infectious diseases; and mental disorders [7]. Beyond 2020 there will not be a standalone adolescent national strategic plan, with adolescent health policy now to be incorporated within a broader strategic plan for reproductive, maternal, newborn, child and adolescent health (RMNCAH) framework. As such, future policy needs to be much more focused and clearly aligned with the key adolescent health needs and drivers of ill health. Alignment is also important in Myanmar given limited resources for health.

A key barrier to the alignment of policy to need in Myanmar has been the relatively limited population health data for adolescents [8]. Administrative data (mortality, hospital and primary level clinic separations) are limited in coverage, with data mostly available through population surveys. Available representative surveys include: the Global School Health Survey (2007 and 2016) [9], Demographic Health Survey (2016) [10], Global Youth Tobacco Survey (2016) [11], and UNICEF’s Multiple Indicator Cluster Survey (2010) [12]. Much of this data is dated, and where available, predominantly focuses on reproductive health and some adolescent health risks, such as tobacco smoking [13,14].

One approach to estimating population disease burden in settings where primary data are limited is to use modelled data, including those available from the Global Burden of Disease study (GBD 2017) [15]. These data build on available population health data and use disease models to estimate mortality, morbidity and health risks across the life course. We have previously used these data to describe adolescent health at a global, regional and national level, serving as a useful tool to inform policy and investment [16–19]. In this paper, we use available primary and modelled data to assemble a comprehensive profile of health needs for adolescents in Myanmar so as to inform policy and actions, and also provide a baseline from which progress can be measured.

## Method

### Stakeholder involvement – governance group

Consistent with best practice in global health research and to ensure appropriate interpretation and translation [20], this work was governed by a group of key stakeholders including: Myanmar Ministry of Health and Sports (MoHS); youth representatives including NGOs engaged in adolescent health; UN agencies (WHO, UNICEF, UNFPA) and donor partners (UNOPS – Access to Health Fund). The group met face to face three times in Yangon on 7 August 2018; 24 January 2019; and 21 February 2020 to inform the framing of the study, interpret the findings and formulate policy recommendations.

### Conceptual framework and data sources

Adolescence was defined as 10 to 24 years, consistent with the neurocognitive, biological and social role transitions that define adolescence [5,21]. This study was framed around the conceptual framework defined in the Lancet Commission of Adolescent Health and Wellbeing (Commission hereafter) [5] to include: health outcomes (mortality, non-fatal diseases and injuries) which are primarily the focus of the health system; health risks (behaviours or states that carry risk for adverse health outcomes in adolescence and into adulthood) which are typically the focus of preventative health interventions; and the structural determinants of health, including social and gender norms.

#### Health outcomes

**Figure 1.**
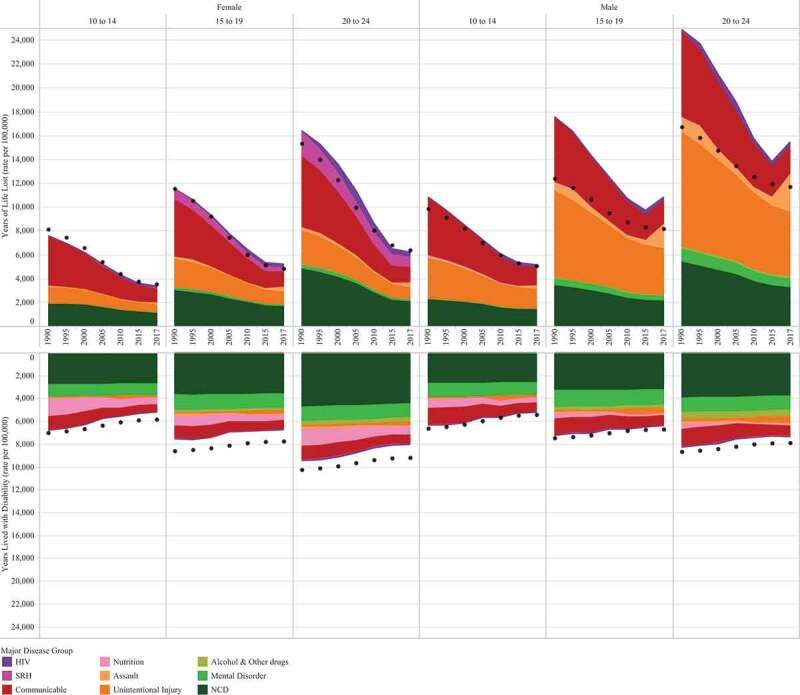
Observed Years of Life Lost and Years Lost to Disability (rate per 100,000) for 10–24-year-olds in Myanmar across 9 categories, by age and sex. Data are from 1990 to 2017 and show overall trends using 5-year slices (the 2008 disaster is not shown). Dotted line indicates all-cause expected YLLs and YLDs. GBD (2017)

Data on health outcomes are drawn from the Global Burden of Diseases Study (GBD) 2017. Methods are described in detail elsewhere [15,22–24], but briefly, GBD 2017 includes a comprehensive and systematic analysis of 359 causes of death, disease and injury. The GBD study gathers the best available primary data and uses a series of disease models to harmonize health estimates and complete missing data. The GBD 2017 estimation process has been thoroughly documented, as well as data sources, in accordance with Guidelines for Accurate and Transparent Health Estimates Reporting (GATHER) [15,22–24]. The causes of death, disease and injury are grouped into four hierarchal levels. Level one has three cause groups reflecting key contributors to the epidemiological transition: communicable, maternal, neonatal, and nutritional disorders; non-communicable diseases; and injuries. Level two has 22 causes, with levels three and four consisting of all disaggregated causes (359). For this analysis individual causes are reported at their most disaggregated level, either level 3 or 4 (see Supplementary Table A2 for complete list). For Figure 1 the disaggregated causes were also regrouped into 9 major groups of relevance to adolescent health, as previously defined by the Commission (see Supplementary Table A1 (summary) & Table A2 (full list)).

#### Health risks

Key health risks were previously identified in the Commission [5]. These were then extended in consultation with the governance group to align with Myanmar’s NSP and available data to include thinness, comprehensive knowledge of HIV prevention, and illicit substance use. Included risks (Supplementary Table A1) broadly related to: nutrition and adult NCD (overweight, underweight, anaemia); sexual reproductive health (SRH) risk (comprehensive knowledge of HIV prevention) and substance use (tobacco, alcohol and illicit substances) [10,25,26]. Data on SRH risk extending beyond knowledge were unavailable. We were also unable to include data on gender-based violence which was requested by the governance group, due to a lack of data. Data on the prevalence of illicit substance use was also limited; here we included attributable disease burden amongst adolescents due to illicit substance use from GBD2017 [26].

#### Health determinants

Health determinants were based on those defined by the Commission [5], and with advice from the governance group we reported additional details on educational attainment and literacy. Health determinants are defined in Supplementary Table A1 and include those relating to education (secondary school completion, quality of education as measured through literacy, and post-secondary transition to employment or training) and SRH (adolescent pregnancy, child marriage and met need for contraception) [10,26,27]. Met need for contraception also serves as an indicator of health service response in the absence of other available data.

### Ethics

Ethics committee approval was not sought for these secondary analyses of openly available, de-identified, datasets.

### Data analysis and reporting

With respect to health outcomes, mortality was reported as both rate and Years of Life Lost (YLL) per population denominator. Non-fatal diseases and injuries is reported as Years of life Lived with a Disability (YLDs), a metric which takes into account prevalence of disease, duration and its severity [15]. We also reported the summary metric of Disability Adjusted Life Years (DALYs), the sum of YLLs and YLDs [24]. Estimates of health outcomes are reported for 2017 (unless specified otherwise), with the corresponding 95% uncertainty interval provided for the top ten individual causes of mortality (Supplementary Table A3), morbidity (Supplementary Table A4), and DALYs (Tables 1 and 2). Uncertainty estimates are distinct from confidence intervals in that they represent uncertainty derived from sampling, model estimation and model specification [24]. Uncertainty intervals were unavailable for the aggregate groupings in Figure 1.

**Table 1.** Top ten causes of DALYs in 10–24-year-old females, 1990 and 2017. Rate (per 100,000) and annualised rate of change (ROC) %, GBD 2017

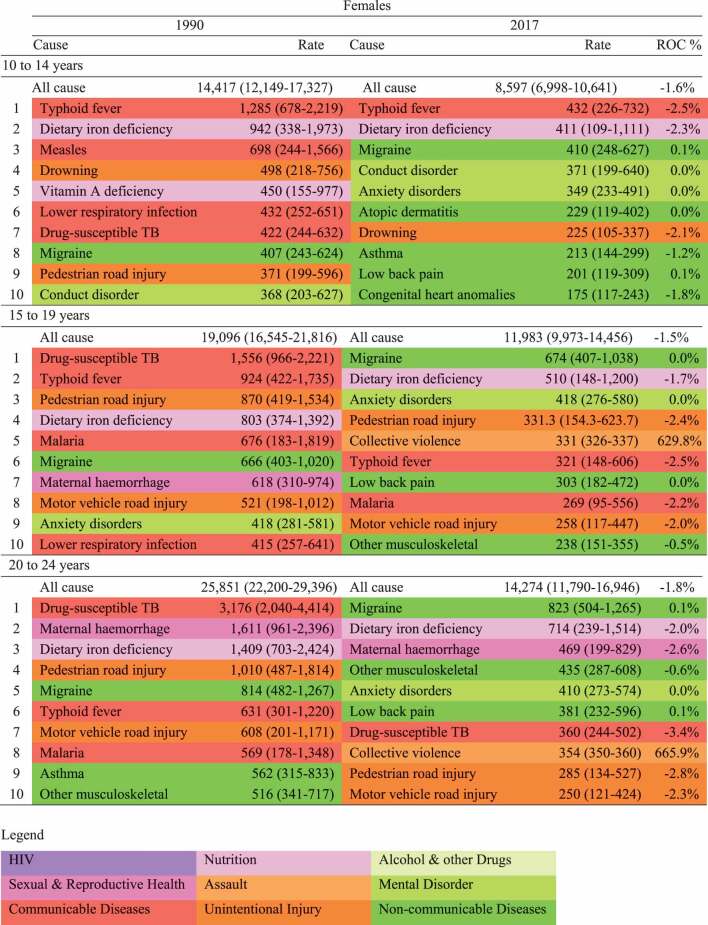

**Table 2.** Top ten causes of DALYs in 10–24-year-old males, 1990 and 2017. Rate (per 100,000) and annualised rate of change (ROC) %, GBD 2017

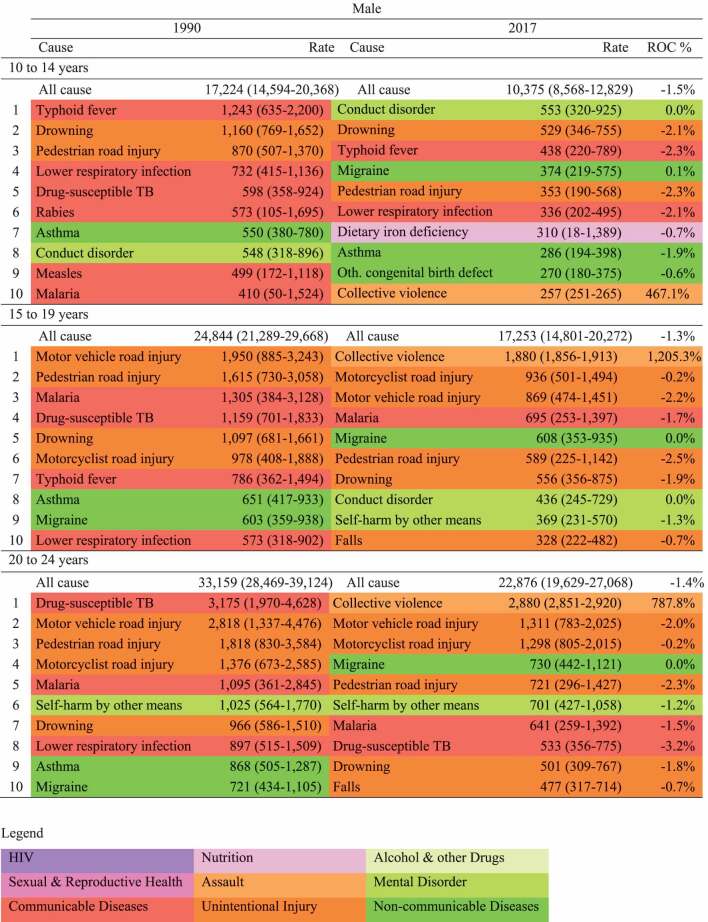

For disaggregated causes of YLLs, YLDs and DALYs, the rate of change since 1990 was reported. Linear regression models were fitted to data points for each cause, and we used the β coefficient to estimate the annual rate of change (ROC, expressed as percentage change), consistent with our previous analyses [19]. Data points for each cause were used from the years 1990, 1995, 2000, 2005, 2010, 2015, 2016 and 2017.

For health outcomes all-cause expected YLLs and YLDs were also included. Expected health outcome trends are an estimate of the rate of YLLs and YLDs given Myanmar’s socio-demographic Index (SDI). SDI is a summary metric derived from income per capita, average years of schooling, and total fertility rate [28]. Throughout this paper where an estimate is referred to be higher, lower, or the same as expected, we are referring to this expected estimate based on SDI.

Health risks and determinants were reported as prevalence (unless otherwise specified). For those estimates sourced from primary data standard errors (and thus 95% confidence intervals) were mostly unavailable. Estimates and uncertainty intervals (where available) for all risks and social determinants, disaggregated by age and sex, are reported in Supplementary Table A5. Where possible, disaggregated data is reported in five-year age bands and by sex.

## Results

### Health outcomes

#### Mortality

In 2017, the all-cause mortality rate for 10–24-year-olds was 71 per 100,000 for females and 149 per 100,000 for males, corresponding to 16,096 adolescent deaths. A higher rate of mortality was observed among the older age groups and among males, with males 20–24 years being at greatest risk (236 per 100,000) (Supplementary Figure A1). Mortality rate has fallen since 1990, however after 2015 the rate plateaued for females, and there was a marked increase for males 15–24 years (Supplementary Figure A1). Overall mortality rates for females were equivalent to those expected based on SDI, but substantially higher for males aged 15–24 years (Figure 1).

Contributors to mortality varied substantially by age and sex (Supplementary Table A3). For 10–14 years, leading contributors were communicable diseases (typhoid and pneumonia), unintentional injuries (drowning and pedestrian injuries) and congenital heart disease. Sex differences were minimal. There was a substantial reduction in rates of cause-specific mortality across categories with the exception of collective violence. For 15–19 year-olds there were important differences; for females, pedestrian accidents, collective violence and typhoid fever were the top three causes, whereas for males, collective violence, motorcycle accidents and motor vehicle accidents were the top three causes, and self-harm was also a major contributor for males. For 20–24-year-olds sex differentials were marked. For females, maternal haemorrhage was the leading cause of death, with collective violence and tuberculosis additional contributors. For males aged 20–24 years, causes of mortality were similar to those aged 15–19 years, but at much higher rates. Of note, whilst HIV related mortality was overall low, it was the only other cause to increase markedly over time. Mortality due to HIV resulting in other diseases in females aged 15–19 years increased from 0.01 per 100,000 in 1990 to 2.2 per 100,000 in 2017, with a similar increase (0.03 to 3.0 per 100,000 in 2017) in 20–24-year-old females.

#### Morbidity

Morbidity for adolescents in Myanmar was overall consistent with expected estimates, with a subtle improvement in burden over time (Figure 1). Key contributors to disease and injury burden were similar across age and sex (Supplementary Table A4); Non-communicable diseases were the overall leading causes, with dietary iron deficiency, neglected tropical diseases (scabies and filariasis) and forces of nature additional contributors. Of the NCDs, chronic pain syndromes (migraine and back and neck pain) were leading causes, with mental disorders (conduct disorders in younger adolescents and anxiety in older adolescents) key drivers. The largest declines in morbidity were seen in the nutritional diseases; females had an annual decline of around 2% for dietary iron deficiency, and whilst declines were less marked than males, the prevalence in 1990 for males was already much lower than their age-respective female counterparts. Most other causes showed only subtle improvements over time, with causes relating to mental health and substance use showing minimal change.

#### Disability-adjusted life years (DALYs)

**Figure 2.**
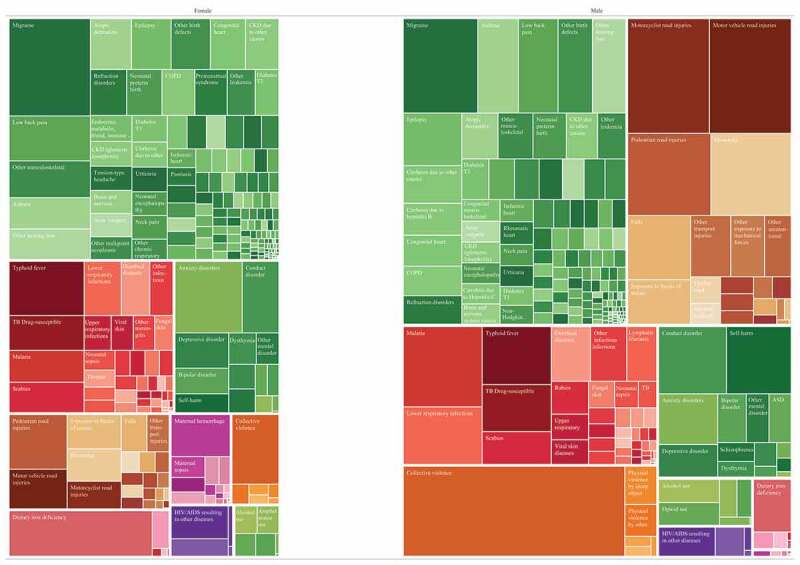
Causes of Disability Adjusted Life Years in 10–24-year-olds by sex in 2017. Each coloured box represents a disaggregated cause, the size of the box is proportional to the DALY rate per 100,000 for that cause. GBD (2017)

Figure 2 presents an overall summary of disease burden for 10–24 years, incorporating both mortality and morbidity (plots for age groups 10–14, 15–19 & 20–24 are in the Supplementary Figures A2 – A4). Overall males have a larger disease burden than females in Myanmar. NCDs are the leading overall cause group for both sexes, however this burden is contributed by multiple smaller causes. Specific leading causes of disease burden are shown in Table 1 for females and Table 2 for males. For females, dietary iron deficiency, migraine and anxiety were common across adolescence, with typhoid a leading cause in 10–14 years and maternal haemorrhage in 20–24-year-olds. For males, conduct disorder, drowning and typhoid were leading causes in 10–14-years, with collective violence and road injuries leading causes for those over the age of 15 years.

### Health risks

**Figure 3.**
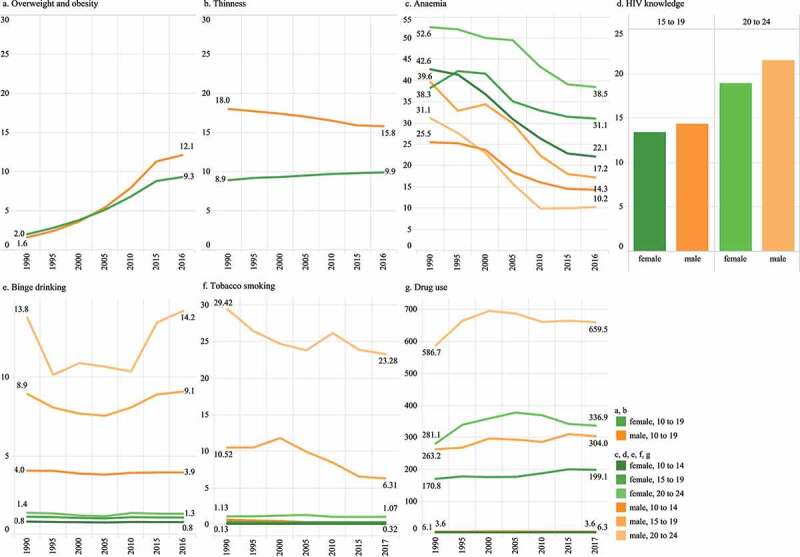
Selected health risks. (a) Prevalence of overweight/obesity among 10–19-year-olds (%) (BMI >+1 SD above the median), WHO (2016). (b) Prevalence of thinness among 10–19-year-olds (%) (BMI <-2 SD below the median), WHO (2016). (c) Prevalence of anaemia among 10–24-year-olds (%), GBD (2017). (d) Comprehensive knowledge of HIV, 15–24-year-olds. DHS (2016). (e) Prevalence of an episode of binge drinking (alcohol >48 g females, >60 g males), 10–24-year-olds, in the past 12mths (%), GBD (2016). (f) Prevalence of tobacco smoking among 10–24-year-olds (%), GBD (2017). (g) DALYs (per 100,000) due to drug use in 10–24-year-olds. GBD (2017)

Of the non-communicable disease risks, under-nutrition and anaemia were highly prevalent in Myanmar (Figure 3(a–c)). The prevalence of overweight and obesity was relatively low (12.1% for males and 9.3% females in 2016), however this was a risk where prevalence has increased markedly over time (Figure 3(a)). Thinness increased slightly for females and declined slightly for males, with males (15.8%) having a higher prevalence than females (9.9%). By contrast females had a greater prevalence of anaemia compared to males, with prevalence generally higher amongst older adolescents (Figure 3(c)). All age and sex groups have seen large reductions in anaemia prevalence, although females 15–19 years had the smallest reduction from 38.3% in 1990 to 31.1.% in 2016.

Risks relating to substance use (Figure 3(e–g)) were substantially more common in males than females. Tobacco smoking was mostly confined to adolescent males over the age of 15 years, with some decline over time. Similarly binge alcohol drinking was a risk mostly of male adolescents, however here adolescent boys aged 10–14 years had a prevalence of 4%. Disease burden due to illicit substance use was largely confined to adolescents over 15 years and higher in males, but also appreciable amongst females, with an increasing trend over time. Comprehensive knowledge of HIV (Figure 3(d)), a measure of SRH risk, showed younger adolescents (15–19 years) and females were less knowledgeable about HIV risks, compared to older and male adolescents.

### Determinants

**Figure 4.**
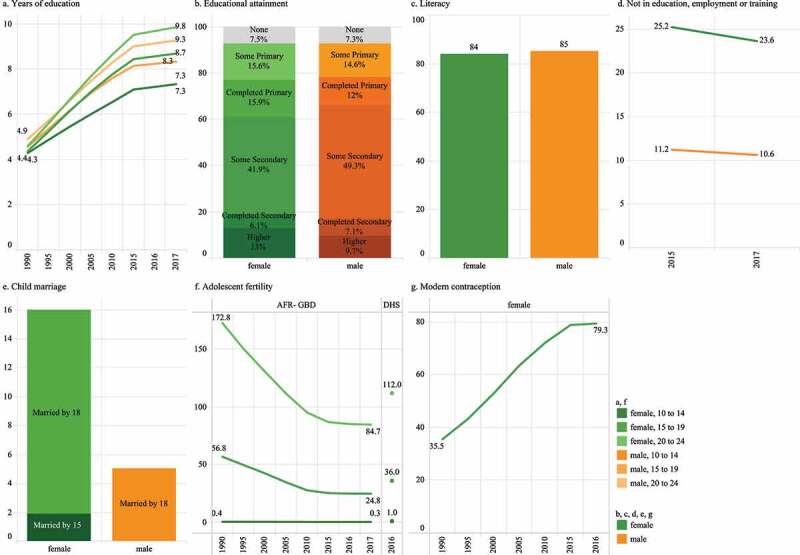
Selected social determinants of health. (a) Mean years of education in 10–24-year-olds, GBD 2017. (b) Educational attainment, 20–24-year-olds (%), DHS 2016. (c) Literacy, 15–24-year-olds (%), UNESCO 2019. (d) Not in education, employment or training, 15–24-years (%), ILO 2019. (e) 20–24-year-olds married before 15 years or 18 years (%), DHS 2016. (f) Live births per 1,000 females, 10–24-years, GBD 2017 & DHS 2016. (g) Demand for modern contraception satisfied, females 15–24-years (%), GBD 2016

Mean years of education for males and females has increased markedly, and in 2016 an estimated 61% of females and 66.1% of male 20–24-year-olds had completed at least some secondary school (Figure 4(a,b)). Literacy (a measure of educational quality; Figure 4(c)) was high for both females and males. However, despite similar literacy and higher secondary school completion, females were twice as likely as males to be not in (further) education, employment or training (23.6% of females in 2019 compared to 10.6% of males; Figure 4(d)). For both sexes the proportion not in education, employment or training has decreased over time.

Available data for child marriage (Figure 4(e)) showed that child marriage mostly occurred among females, and between the ages of 15 and 18 years. Adolescent pregnancy among young adolescent girls was low, with current primary estimates of 1 per 1,000 for 10–14-year-olds, 36 for 15–19-year-olds, and 112 for 20–24-year-olds; available modelled data demonstrate substantial reductions over time (Figure 4(f)). In that same period, demand for contraception satisfied with modern methods for 15–24-year-olds has increased from 35% in 1990 to 79% in 2016 (Figure 4(g)).

## Discussion

Our study revealed considerable changes in the health of Myanmar’s adolescents over the past 25 years. Despite improvements since 1990, a substantial burden of communicable, maternal and nutritional disease persists. There is a large and unshifting burden of injuries and accidents, contributing substantially to mortality; motorcycle injuries remain high and there have been recent increases in harms due to collective violence. There is also a large and mostly unchanging burden of non-communicable diseases (NCDs), including mental disorder. These shifts in disease burden have resulted in NCDs being the dominant contributor to disease burden for females and injuries for males. For males, this has resulted in a mortality rate that is in excess of that expected based on Myanmar’s SDI. Health risks relating to substance use and body mass are relatively low (although there is a trend of these increasing), with indicators of under nutrition (underweight and anaemia) persisting. Health determinants relating to adolescent pregnancy, child marriage and access to modern contraception are favourable, and there is evidence of good progress in education. However, transition from secondary education to employment and further training is incomplete, especially for females. In the absence of comprehensive primary data, these largely modelled estimates provide important guidance around priority areas for health policy, and where data collection efforts should be strengthened.

The findings of this analysis are consistent with previous global analyses using modelled data where Myanmar has been defined as ‘multi-burden’ country for adolescent health outcomes (large burden of disease across the epidemiological transition) [19]. The findings are also consistent with the limited available primary data from Myanmar and the South-east Asia region that mostly focusses on health risks and determinants for adolescents. Data from the Global School Health Survey show Myanmar to have comparatively lower rates of obesity (10.7%) compared to South-east Asian countries like Thailand (20.4%) [25,29]. Social determinants such as child marriage and adolescent fertility in Myanmar are also favourable [19]; 35.4% of adult women in Laos and 22.5% in Thailand were married before the age of 18 years, compared to 16% in Myanmar [30]. Adolescent births in Myanmar (17 per 1,000 15–19-year-olds) are also considerably lower than in Laos (94 per 1,000) and Thailand (60 per 1,000) [30].

The profile of health needs for adolescents in Myanmar is relatively aligned with policy efforts to date. Indeed, all seven priority areas of Myanmar’s adolescent National Strategic Plan (sexual and reproductive health; HIV; nutrition; substance use; unintentional injuries; infectious diseases; and mental disorders) are important contributors to disease and injury. However, there are some important contributors to poor health that are not current targets of adolescent health policy in Myanmar. For example, collective violence has recently emerged as an important contributor to mortality for adolescents in Myanmar, especially for males. It is important to note that these estimates are based on a high degree of modelling (see Tables 1 and 2 and in Supplementary Tables A3 and A4 for uncertainty estimates), however they appear relatively consistent with WHO data for SDG target 16.1.2 (deaths from conflict) [31]. Conflict in any setting, in addition to acute mortality, risks reversing gains made in health care and development [16]. Health system strengthening can be an apolitical means of promoting peace through reducing inequalities and promoting social justice [32]. Further, health systems can help promote peace through building trust between the government and local ethnic communities [33].

Non-communicable diseases also emerged as an important contributor of poor health that was not well reflected in policy and action. Key contributors to NCD burden for adolescents in Myanmar were mental disorder (anxiety, conduct disorder, self-harm), asthma, migraine, and low back pain. Further, risk factors associated with NCD have also increased, consistent with trends in other low and middle income settings [19]. The 2014 national WHO STEPs survey of NCD risk in adults in Myanmar showed around that the majority of 25–44-year-olds had at least one NCD risk factor, and 15% had 3 to 5 NCD risk factors, with these risks all increasing in prevalence since 2009 [34]. Adolescents are often overlooked in efforts to control NCDs, however the findings of our analysis reinforce the need for equitable access to quality screening and health [35], particularly around mental health, given the burden of mental disorder for adolescents of both sexes in Myanmar.

The policy focus in the current National Strategic Plan around communicable diseases in adolescence has been on malaria, dengue and tuberculosis. Our findings confirm there has been progress around malaria and TB, however both remain as significant contributors to disease burden for adolescents; dengue has increased in burden however it is not amongst the leading causes for adolescents. In addition, we also found that typhoid and scabies were important outcomes, but not well reflected in policy. Our findings are consistent with a recent study finding high ongoing incidence of typhoid and paratyphoid amongst adolescents and adults in Yangon [36]. Scabies has also been identified as contributing to burden of disease in Myanmar in other studies using GBD data [37], however further studies show there is a lack of primary data underpinning the modelled data for scabies, indicating a data gap in this area [38].

Findings related to sexual and reproductive health- a dominant focus of adolescent policy in Myanmar and globally- were mixed. Some indicators of SRH – particularly child marriage and adolescent pregnancy were favourable. However, maternal mortality was one of the leading causes of mortality in 15–19-, and the leading cause in 20–24-year-olds females, consistent with data from comparable settings [39]. We also found HIV knowledge to be low, especially among females. These findings are concerning, given HIV-related mortality was one of the few causes of death which had increased, particularly in females. There were other areas of SRH, particularly related to gender and menstrual health that are not well reflected in this analysis. Our findings suggest that sexual and reproductive health should remain a focus of future policy, especially in the context of rapid sociodemographic change and the emergence of new sexual health risks.

Our findings also show adolescence is where many gender disparities become apparent, consistent with a recent analysis for the Asia Pacific region [40]. Females appeared disadvantaged in some determinants of health (post-educational opportunities and child marriage), while males had a higher prevalence of health risk behaviours, particularly related to substance use. There were also large gender differentials in health outcome not necessarily driven by biological sex; females aged 20–24 years had a larger burden of HIV and nutritional disorders, with males having an excess burden of morbidity and mortality, driven largely by injury. Socially constructed gender norms impact roles and opportunities for all people, influencing all aspects of health and wellbeing including the determinants of health and access to quality healthcare [41]. It is likely that the same underlying gender norms that reward dominant constructs of masculinity and contribute to boys’ risk-taking behaviour are also limiting girls’ opportunities and agency [40].

Collectively, these findings highlight the need to focus future efforts on health outcomes (diseases and injuries) affecting adolescents, whilst maintaining a focus on preventing health risks and assuring the determinants of health. Adolescents are often overlooked in health service response; however, the findings of this analysis challenge the misconception that they are without health needs [42]. Adolescent friendly health services (AFHS) can play an important role in preventing and responding to the health needs of adolescents, a strengthening and extension of AFHS in Myanmar would be key to tackling preventable morbidity and mortality [42]. School and other educational facilities provide a particularly important setting for prevention and response to health needs. Local school-based interventions can provide cost-effective, needs-based, equitable responses to these key areas of need (preventable injuries, NCDs, and mental health), to improve health literacy, or provide direct access to adolescent-friendly health services [5,43].

The strength of this work lies in the partnership with key stakeholders, including Government and UN agencies. This ensures that the work is aligned to needs, appropriately interpreted, and is also enabling of translation to action. Given the paucity of primary data we have relied heavily on modelled estimates, particularly so for morbidity and mortality. These modelled estimates are based on what primary data are available, for both outcomes of interest and covariates, including primary data from countries and regions that are similar geographically or in stage of development [15,22–24]. For many estimates uncertainty is substantial. Nonetheless, these data provide a guide to where policy and action should be focussed (in the absence of any other data) and also highlight where data collection efforts should be focussed. There are other key health issues critical to this life-phase, such as gender-based violence, or menstrual health and hygiene, that we could not include given we were bound to the Global Burden of Disease list of causes. Data on determinants of health were limited, and we could also not disaggregate health estimates by wealth, ethnicity, education, or urban/rural status. With improved data availability this should be a focus of future work.

## Conclusion

Our analysis highlights that the health needs for adolescents in Myanmar have changed substantially over the past 25 years. Progress has perhaps been most marked in addressing health risks and determinants, with progress in health outcomes comparatively lagging. As such, these data highlight the need to focus current efforts on addressing disease and mortality experienced by adolescents in Myanmar. The priorities identified here can not only help to focus policy investments in adolescent health, but also data collection efforts and future research.

## Supplementary Material

Supplemental Material

## Data Availability

The data that support the findings of this study are openly available in an online repository, references and weblinks available in supplementary material Table A1.

## Paper context

Rapid epidemiological transition in Myanmar has resulted in complex health needs for adolescents. Limited population health data for adolescents has been a barrier to the alignment of policy to need. We found a large emergent burden of injuries and non-communicable diseases in the context of ongoing communicable, nutritional and reproductive health needs. To have the greatest impact on death and disability, adolescent health policies should now focus on investments which target non-communicable disease and injuries.
