# Supplementary material for: Matching action to need: an analysis of Global Burden of Disease 2017 and population health data to focus adolescent health policy and actions in Myanmar
Source: Glob Health Action. 2021 Jan 15;14(1):1844976. doi: 10.1080/16549716.2020.1844976 (PMC7833024; doi:10.1080/16549716.2020.1844976)
Supplement: Supplemental Material [file ZGHA_A_1844976_SM8888.pdf]

Table A1. Health outcome groupings and indicator definitions for health risks and determinants.

Indicator	Age	Definition	Data source	Year
Health outcomes				
Major grouping	10 – 24	*Including but not limited to the following causes:*		
HIV		HIV/AIDS and HIV/AIDS resulting in other diseases.	Global Burden of Disease (GBD) Study [1]	2017
Sexual and reproductive health		STIs and maternal disorders.		
Communicable diseases		Encephalitis, meningitis, tuberculosis, typhoid, neglected tropical diseases, vaccine preventable diseases, diarrheal diseases, hepatitis, malaria, neonatal sepsis, respiratory infections, communicable skin diseases.		
Nutrition		Iron deficiency anaemia and nutritional deficiency.		
Assault		physical violence by firearm, by sharp object, sexual violence, conflict and terrorism, collective violence.		
Unintentional injury		Animal injury, drowning, falls, fire, forces of nature, foreign body injury, mechanical forces injury, poisoning, transport injury.		
Alcohol and other Drugs		Substance use disorders of alcohol, opioid, cocaine, amphetamine, and cannabis.		
Mental disorder		Intellectual disability, anxiety, behavioural disorder, bipolar, depression, eating disorder, psychotic disorders, self-harm		
Non-communicable Diseases		Arthritis, asthma, low back or neck pain, cardiovascular diseases, congenital, dental, diabetes, endocrine, epilepsy, gastrointestinal, gynaecological, haemoglobinopathy, migraine, neoplasm, neurological, other musculoskeletal, renal, respiratory, sensory disorders, non-communicable skin diseases.		
Health risks				
Overweight and obesity	10 – 19	BMI measured >1 standard deviation above the median estimate (%)	WHO Global Health Observatory [2]	2019
Thinness	10 – 19	BMI <2 standard deviation below median estimate (%)	WHO Global Health Observatory [2]	2019
Anaemia	10 – 14	Males and females: haemoglobin <115 g/L (%)	GBD study [1]	2017
	15 – 24	males: haemoglobin <130 g/L; non-pregnant females: haemoglobin <120 g/L; pregnant females: haemoglobin <110 g/L (%)		
Comprehensive knowledge of HIV	15 – 24	Percentage of 15-24-year-olds with comprehensive knowledge of HIV (who correctly identify the two ways of preventing the sexual transmission of HIV, who know that a healthy-looking person can be HIV-positive and who reject the two most common misconceptions about HIV transmission)	Myanmar DHS [3]	2015-2016

Tobacco smoking	10 – 24	Current tobacco smoking (%), by 5-year group	GBD study [1]	2017
Binge drinking	15 – 24	Males: >60 grams of alcohol on single occasion in the last 12 months (%)<br>Females: >48 grams of alcohol on single occasion in the last 12 months (%)	GBD study [1]	2016
Illicit drug use	10 – 24	All-cause DALY rate attributed to illicit drug use	GBD study [1]	2017
Determinants				
Literacy	15 – 24	Percentage of literate persons (%)	UNESCO Institute for Statistics [4]	2019
Mean years of education	10 – 24	Mean number of years of education attainment, age specific.	GBD study [1]	2017
Educational Attainment	20 – 24	Percent distribution of 20-24-year-olds by highest level of schooling attended or completed.	Myanmar DHS [3]	2015-2016
Not in employment, education, or training	15 – 24	Share of youth not in employment, education, or training (%)	International Labour Organization [5]	2019
Child Marriage	20 – 24	Adolescents married before age 15; and before age 18 (%)	Myanmar DHS [3]	2015-2016
Adolescent fertility rate	10 – 24	Live births per 1,000 females aged 10-24-years, in 5-year age bands	GBD study [1]	2017
Adolescent fertility rate	10 – 24	Live births per 1,000 females aged 10-24-years, in 5-year age bands	Myanmar DHS [3]	2015-2016
Met need for contraception	15 – 24	Proportion of females aged 15-24-years who have their need for family planning satisfied with modern methods (%)	GBD study [1]	2016

References
[1] GBD Study 2017. Global Burden of Disease Study 2017 (GBD 2017) Results. Seattle, United States: Institute for Health Metrics and Evaluation (IHME), 2018. Available at: http://ghdx.healthdata.org/gbd-results-tool [Acessed 21st November 2018].

[2] World Health Organization. Global Health Observatory (GHO) data [online database]. Available at: https://apps.who.int/gho/data/node.home [Accessed 04 March 2019].

[3] Ministry of Health and Sports - MoHS/Myanmar and ICF. Myanmar Demographic and Health Survey 2015-16. Nay Pyi Taw, Myanmar: MoHS and ICF, 2017. Available at: http://dhsprogram.com/pubs/pdf/FR324/FR324.pdf [Accessed 10 February 2020].

[4] UNESCO Institute of Statistics. UNESCO Institute of Statistics: Myanmar statistics. Available at: http://uis.unesco.org/en/country/mm [Accessed 1 March 2020].

[5] International Labour Organization. ILOSTAT database [database]. 2020. Available at: https://ilostat.ilo.org/data/ [Accessed 05 April 2019].

Table A2. Full list of GBD 2017 disaggregated causes and their corresponding major group of relevance to adolescent health.

GBD 2017 Cause ID	GBD 2017 Cause name	Level	Major Group
948	HIV/AIDS - Drug-susceptible Tuberculosis	4	HIV
949	HIV/AIDS - Multidrug-resistant Tuberculosis without extensive drug resistance	4	HIV
950	HIV/AIDS - Extensively drug-resistant Tuberculosis	4	HIV
300	HIV/AIDS resulting in other diseases	4	HIV
394	Syphilis	4	Sexual & reproductive health
395	Chlamydial infection	4	Sexual & reproductive health
396	Gonococcal infection	4	Sexual & reproductive health
397	Trichomoniasis	4	Sexual & reproductive health
398	Genital herpes	4	Sexual & reproductive health
399	Other sexually transmitted infections	4	Sexual & reproductive health
367	Maternal hemorrhage	4	Sexual & reproductive health
368	Maternal sepsis and other maternal infections	4	Sexual & reproductive health
369	Maternal hypertensive disorders	4	Sexual & reproductive health
370	Maternal obstructed labor and uterine rupture	4	Sexual & reproductive health
995	Maternal abortion and miscarriage	4	Sexual & reproductive health
374	Ectopic pregnancy	4	Sexual & reproductive health
375	Indirect maternal deaths	4	Sexual & reproductive health
376	Late maternal deaths	4	Sexual & reproductive health
741	Maternal deaths aggravated by HIV/AIDS	4	Sexual & reproductive health
379	Other maternal disorders	4	Sexual & reproductive health
954	Latent tuberculosis infection	4	Communicable diseases
934	Drug-susceptible tuberculosis	4	Communicable diseases
946	Multidrug-resistant tuberculosis without extensive drug resistance	4	Communicable diseases
947	Extensively drug-resistant tuberculosis	4	Communicable diseases
322	Lower respiratory infections	3	Communicable diseases
328	Upper respiratory infections	3	Communicable diseases
329	Otitis media	3	Communicable diseases
302	Diarrheal diseases	3	Communicable diseases
319	Typhoid fever	4	Communicable diseases
320	Paratyphoid fever	4	Communicable diseases

959	Invasive Non-typhoidal Salmonella (iNTS)	3	Communicable diseases
321	Other intestinal infectious diseases	3	Communicable diseases
345	Malaria	3	Communicable diseases
346	Chagas disease	3	Communicable diseases
348	Visceral leishmaniasis	4	Communicable diseases
349	Cutaneous and mucocutaneous leishmaniasis	4	Communicable diseases
350	African trypanosomiasis	3	Communicable diseases
351	Schistosomiasis	3	Communicable diseases
352	Cysticercosis	3	Communicable diseases
353	Cystic echinococcosis	3	Communicable diseases
354	Lymphatic filariasis	3	Communicable diseases
355	Onchocerciasis	3	Communicable diseases
356	Trachoma	3	Communicable diseases
357	Dengue	3	Communicable diseases
358	Yellow fever	3	Communicable diseases
359	Rabies	3	Communicable diseases
361	Ascariasis	4	Communicable diseases
362	Trichuriasis	4	Communicable diseases
363	Hookworm disease	4	Communicable diseases
364	Food-borne trematodiases	3	Communicable diseases
405	Leprosy	3	Communicable diseases
843	Ebola	3	Communicable diseases
935	Zika virus	3	Communicable diseases
936	Guinea worm disease	3	Communicable diseases
365	Other neglected tropical diseases	3	Communicable diseases
333	Pneumococcal meningitis	4	Communicable diseases
334	H influenzae type B meningitis	4	Communicable diseases
335	Meningococcal meningitis	4	Communicable diseases
336	Other meningitis	4	Communicable diseases
337	Encephalitis	3	Communicable diseases
338	Diphtheria	3	Communicable diseases
339	Whooping cough	3	Communicable diseases

340	Tetanus	3	Communicable diseases
341	Measles	3	Communicable diseases
342	Varicella and herpes zoster	3	Communicable diseases
401	Acute hepatitis A	4	Communicable diseases
402	Acute hepatitis B	4	Communicable diseases
403	Acute hepatitis C	4	Communicable diseases
404	Acute hepatitis E	4	Communicable diseases
408	Other unspecified infectious diseases	3	Communicable diseases
383	Neonatal sepsis and other neonatal infections	4	Communicable diseases
656	Cellulitis	4	Communicable diseases
657	Pyoderma	4	Communicable diseases
658	Scabies	3	Communicable diseases
659	Fungal skin diseases	3	Communicable diseases
660	Viral skin diseases	3	Communicable diseases
387	Protein-energy malnutrition	3	Nutrition
388	Iodine deficiency	3	Nutrition
389	Vitamin A deficiency	3	Nutrition
390	Dietary iron deficiency	3	Nutrition
391	Other nutritional deficiencies	3	Nutrition
381	Neonatal preterm birth	4	Non-communicable diseases
382	Neonatal encephalopathy due to birth asphyxia and trauma	4	Non-communicable diseases
384	Hemolytic disease and other neonatal jaundice	4	Non-communicable diseases
385	Other neonatal disorders	4	Non-communicable diseases
444	Lip and oral cavity cancer	3	Non-communicable diseases
447	Nasopharynx cancer	3	Non-communicable diseases
450	Other pharynx cancer	3	Non-communicable diseases
411	Esophageal cancer	3	Non-communicable diseases
414	Stomach cancer	3	Non-communicable diseases
441	Colon and rectum cancer	3	Non-communicable diseases
418	Liver cancer due to hepatitis B	4	Non-communicable diseases
419	Liver cancer due to hepatitis C	4	Non-communicable diseases
420	Liver cancer due to alcohol use	4	Non-communicable diseases

996	Liver cancer due to NASH	4	Non-communicable diseases
421	Liver cancer due to other causes	4	Non-communicable diseases
453	Gallbladder and biliary tract cancer	3	Non-communicable diseases
456	Pancreatic cancer	3	Non-communicable diseases
423	Larynx cancer	3	Non-communicable diseases
426	Tracheal, bronchus, and lung cancer	3	Non-communicable diseases
459	Malignant skin melanoma	3	Non-communicable diseases
849	Non-melanoma skin cancer (squamous-cell carcinoma)	4	Non-communicable diseases
850	Non-melanoma skin cancer (basal-cell carcinoma)	4	Non-communicable diseases
429	Breast cancer	3	Non-communicable diseases
432	Cervical cancer	3	Non-communicable diseases
435	Uterine cancer	3	Non-communicable diseases
465	Ovarian cancer	3	Non-communicable diseases
438	Prostate cancer	3	Non-communicable diseases
468	Testicular cancer	3	Non-communicable diseases
471	Kidney cancer	3	Non-communicable diseases
474	Bladder cancer	3	Non-communicable diseases
477	Brain and nervous system cancer	3	Non-communicable diseases
480	Thyroid cancer	3	Non-communicable diseases
483	Mesothelioma	3	Non-communicable diseases
484	Hodgkin lymphoma	3	Non-communicable diseases
485	Non-Hodgkin lymphoma	3	Non-communicable diseases
486	Multiple myeloma	3	Non-communicable diseases
845	Acute lymphoid leukemia	4	Non-communicable diseases
846	Chronic lymphoid leukemia	4	Non-communicable diseases
847	Acute myeloid leukemia	4	Non-communicable diseases
848	Chronic myeloid leukemia	4	Non-communicable diseases
943	Other leukemia	4	Non-communicable diseases
489	Other malignant neoplasms	3	Non-communicable diseases
964	Myelodysplastic, myeloproliferative, and other hematopoietic neoplasms	4	Non-communicable diseases
965	Benign and in situ intestinal neoplasms	4	Non-communicable diseases
966	Benign and in situ cervical and uterine neoplasms	4	Non-communicable diseases

967	Other benign and in situ neoplasms	4	Non-communicable diseases
492	Rheumatic heart disease	3	Non-communicable diseases
493	Ischemic heart disease	3	Non-communicable diseases
495	Ischemic stroke	4	Non-communicable diseases
496	Intracerebral hemorrhage	4	Non-communicable diseases
497	Subarachnoid hemorrhage	4	Non-communicable diseases
498	Hypertensive heart disease	3	Non-communicable diseases
968	Non-rheumatic calcific aortic valve disease	4	Non-communicable diseases
969	Non-rheumatic degenerative mitral valve disease	4	Non-communicable diseases
970	Other non-rheumatic valve diseases	4	Non-communicable diseases
942	Myocarditis	4	Non-communicable diseases
938	Alcoholic cardiomyopathy	4	Non-communicable diseases
944	Other cardiomyopathy	4	Non-communicable diseases
500	Atrial fibrillation and flutter	3	Non-communicable diseases
501	Aortic aneurysm	3	Non-communicable diseases
502	Peripheral artery disease	3	Non-communicable diseases
503	Endocarditis	3	Non-communicable diseases
507	Other cardiovascular and circulatory diseases	3	Non-communicable diseases
509	Chronic obstructive pulmonary disease	3	Non-communicable diseases
511	Silicosis	4	Non-communicable diseases
512	Asbestosis	4	Non-communicable diseases
513	Coal workers pneumoconiosis	4	Non-communicable diseases
514	Other pneumoconiosis	4	Non-communicable diseases
515	Asthma	3	Non-communicable diseases
516	Interstitial lung disease and pulmonary sarcoidosis	3	Non-communicable diseases
520	Other chronic respiratory diseases	3	Non-communicable diseases
522	Cirrhosis and other chronic liver diseases due to hepatitis B	4	Non-communicable diseases
523	Cirrhosis and other chronic liver diseases due to hepatitis C	4	Non-communicable diseases
524	Cirrhosis and other chronic liver diseases due to alcohol use	4	Non-communicable diseases
971	Cirrhosis due to NASH	4	Non-communicable diseases
525	Cirrhosis and other chronic liver diseases due to other causes	4	Non-communicable diseases
527	Peptic ulcer disease	4	Non-communicable diseases

528	Gastritis and duodenitis	4	Non-communicable diseases
536	Gastroesophageal reflux disease	4	Non-communicable diseases
529	Appendicitis	3	Non-communicable diseases
530	Paralytic ileus and intestinal obstruction	3	Non-communicable diseases
531	Inguinal, femoral, and abdominal hernia	3	Non-communicable diseases
532	Inflammatory bowel disease	3	Non-communicable diseases
533	Vascular intestinal disorders	3	Non-communicable diseases
534	Gallbladder and biliary diseases	3	Non-communicable diseases
535	Pancreatitis	3	Non-communicable diseases
541	Other digestive diseases	3	Non-communicable diseases
543	Alzheimer's disease and other dementias	3	Non-communicable diseases
544	Parkinson's disease	3	Non-communicable diseases
545	Epilepsy	3	Non-communicable diseases
546	Multiple sclerosis	3	Non-communicable diseases
554	Motor neuron disease	3	Non-communicable diseases
547	Migraine	4	Non-communicable diseases
548	Tension-type headache	4	Non-communicable diseases
557	Other neurological disorders	3	Non-communicable diseases
975	Diabetes mellitus type 1	4	Non-communicable diseases
976	Diabetes mellitus type 2	4	Non-communicable diseases
997	Chronic kidney disease due to diabetes mellitus type 1	4	Non-communicable diseases
998	Chronic kidney disease due to diabetes mellitus type 2	4	Non-communicable diseases
591	Chronic kidney disease due to hypertension	4	Non-communicable diseases
592	Chronic kidney disease due to glomerulonephritis	4	Non-communicable diseases
593	Chronic kidney disease due to other and unspecified causes	4	Non-communicable diseases
588	Acute glomerulonephritis	3	Non-communicable diseases
977	Atopic dermatitis	4	Non-communicable diseases
978	Contact dermatitis	4	Non-communicable diseases
979	Seborrhoeic dermatitis	4	Non-communicable diseases
655	Psoriasis	3	Non-communicable diseases
661	Acne vulgaris	3	Non-communicable diseases
662	Alopecia areata	3	Non-communicable diseases

663	Pruritus	3	Non-communicable diseases
664	Urticaria	3	Non-communicable diseases
665	Decubitus ulcer	3	Non-communicable diseases
668	Other skin and subcutaneous diseases	3	Non-communicable diseases
670	Glaucoma	4	Non-communicable diseases
671	Cataract	4	Non-communicable diseases
672	Age-related macular degeneration	4	Non-communicable diseases
999	Refraction disorders	4	Non-communicable diseases
1000	Near vision loss	4	Non-communicable diseases
675	Other vision loss	4	Non-communicable diseases
674	Age-related and other hearing loss	3	Non-communicable diseases
679	Other sense organ diseases	3	Non-communicable diseases
627	Rheumatoid arthritis	3	Non-communicable diseases
628	Osteoarthritis	3	Non-communicable diseases
630	Low back pain	3	Non-communicable diseases
631	Neck pain	3	Non-communicable diseases
632	Gout	3	Non-communicable diseases
639	Other musculoskeletal disorders	3	Non-communicable diseases
642	Neural tube defects	4	Non-communicable diseases
643	Congenital heart anomalies	4	Non-communicable diseases
644	Orofacial clefts	4	Non-communicable diseases
645	Down syndrome	4	Non-communicable diseases
646	Turner syndrome	4	Non-communicable diseases
647	Klinefelter syndrome	4	Non-communicable diseases
648	Other chromosomal abnormalities	4	Non-communicable diseases
649	Congenital musculoskeletal and limb anomalies	4	Non-communicable diseases
650	Urogenital congenital anomalies	4	Non-communicable diseases
651	Digestive congenital anomalies	4	Non-communicable diseases
652	Other congenital birth defects	4	Non-communicable diseases
595	Urinary tract infections	4	Non-communicable diseases
596	Urolithiasis	4	Non-communicable diseases
597	Benign prostatic hyperplasia	4	Non-communicable diseases

598	Male infertility	4	Non-communicable diseases
602	Other urinary diseases	4	Non-communicable diseases
604	Uterine fibroids	4	Non-communicable diseases
605	Polycystic ovarian syndrome	4	Non-communicable diseases
606	Female infertility	4	Non-communicable diseases
607	Endometriosis	4	Non-communicable diseases
608	Genital prolapse	4	Non-communicable diseases
609	Premenstrual syndrome	4	Non-communicable diseases
612	Other gynecological diseases	4	Non-communicable diseases
614	Thalassemias	4	Non-communicable diseases
837	Thalassemias trait	4	Non-communicable diseases
615	Sickle cell disorders	4	Non-communicable diseases
838	Sickle cell trait	4	Non-communicable diseases
616	G6PD deficiency	4	Non-communicable diseases
839	G6PD trait	4	Non-communicable diseases
618	Other hemoglobinopathies and hemolytic anemias	4	Non-communicable diseases
619	Endocrine, metabolic, blood, and immune disorders	3	Non-communicable diseases
681	Caries of deciduous teeth	4	Non-communicable diseases
682	Caries of permanent teeth	4	Non-communicable diseases
683	Periodontal diseases	4	Non-communicable diseases
684	Edentulism and severe tooth loss	4	Non-communicable diseases
685	Other oral disorders	4	Non-communicable diseases
686	Sudden infant death syndrome	3	Non-communicable diseases
559	Schizophrenia	3	Mental disorder
568	Major depressive disorder	4	Mental disorder
569	Dysthymia	4	Mental disorder
570	Bipolar disorder	3	Mental disorder
571	Anxiety disorders	3	Mental disorder
573	Anorexia nervosa	4	Mental disorder
574	Bulimia nervosa	4	Mental disorder
575	Autism spectrum disorders	3	Mental disorder
578	Attention-deficit/hyperactivity disorder	3	Mental disorder

579	Conduct disorder	3	Mental disorder
582	Idiopathic developmental intellectual disability	3	Mental disorder
585	Other mental disorders	3	Mental disorder
721	Self-harm by firearm	4	Mental disorder
723	Self-harm by other specified means	4	Mental disorder
560	Alcohol use disorders	3	Alcohol & other drugs
562	Opioid use disorders	4	Alcohol & other drugs
563	Cocaine use disorders	4	Alcohol & other drugs
564	Amphetamine use disorders	4	Alcohol & other drugs
565	Cannabis use disorders	4	Alcohol & other drugs
566	Other drug use disorders	4	Alcohol & other drugs
690	Pedestrian road injuries	4	Unintentional Injury
691	Cyclist road injuries	4	Unintentional Injury
692	Motorcyclist road injuries	4	Unintentional Injury
693	Motor vehicle road injuries	4	Unintentional Injury
694	Other road injuries	4	Unintentional Injury
695	Other transport injuries	3	Unintentional Injury
697	Falls	3	Unintentional Injury
698	Drowning	3	Unintentional Injury
699	Fire, heat, and hot substances	3	Unintentional Injury
701	Poisoning by carbon monoxide	4	Unintentional Injury
703	Poisoning by other means	4	Unintentional Injury
705	Unintentional firearm injuries	4	Unintentional Injury
707	Other exposure to mechanical forces	4	Unintentional Injury
708	Adverse effects of medical treatment	3	Unintentional Injury
710	Venomous animal contact	4	Unintentional Injury
711	Non-venomous animal contact	4	Unintentional Injury
713	Pulmonary aspiration and foreign body in airway	4	Unintentional Injury
714	Foreign body in eyes	4	Unintentional Injury
715	Foreign body in other body part	4	Unintentional Injury
842	Environmental heat and cold exposure	3	Unintentional Injury
729	Exposure to forces of nature	3	Unintentional Injury

716	Other unintentional injuries	3	Unintentional Injury
725	Physical violence by firearm	4	Assault
726	Physical violence by sharp object	4	Assault
941	Sexual violence	4	Assault
727	Physical violence by other means	4	Assault
945	Conflict and terrorism	3	Assault
854	Executions and police conflict	3	Assault

Figure A1. All-cause mortality rate (/100,000 pa) by sex, 10-24 years, 1990-2017

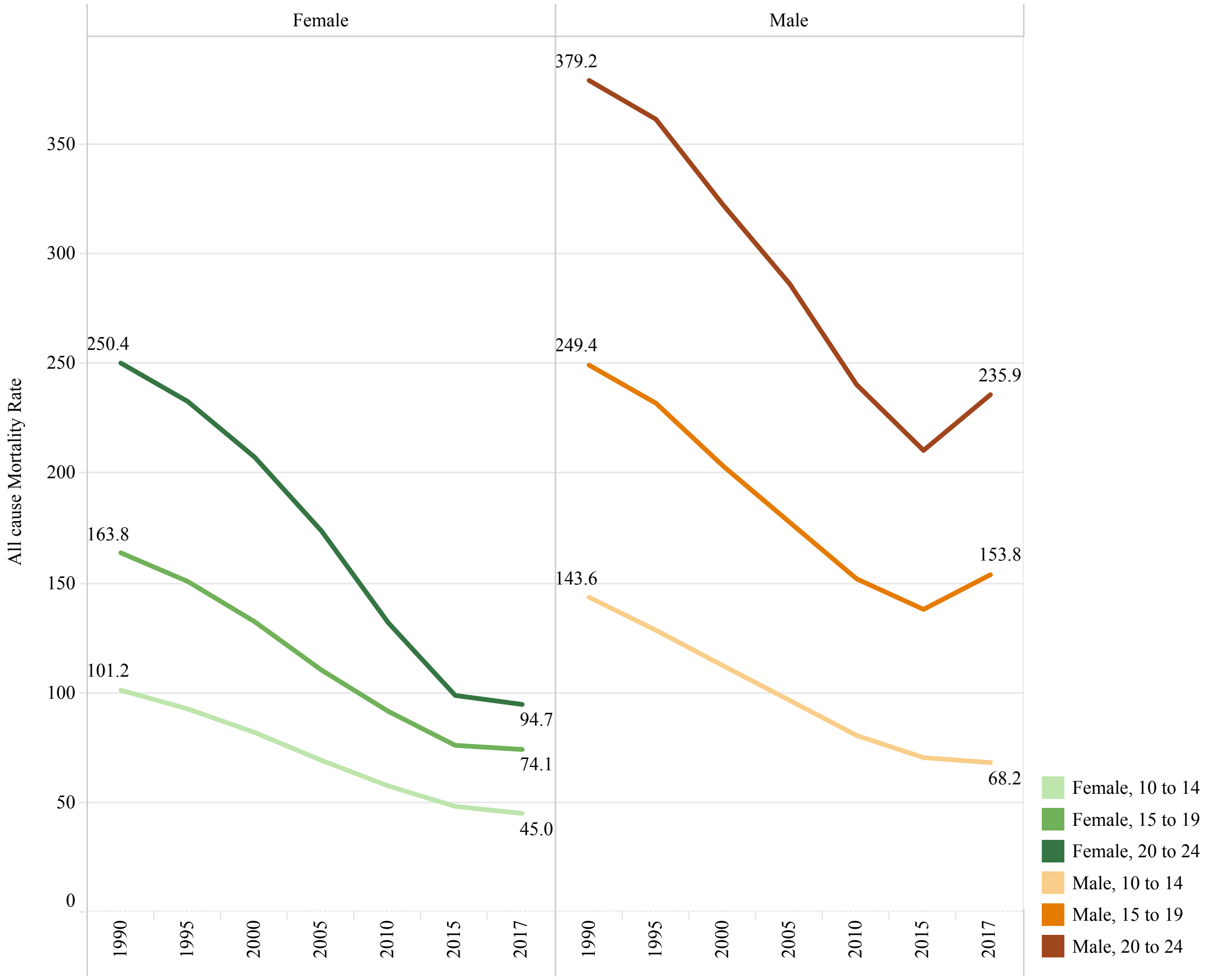

Table A3. Top ten causes of Mortality in 10-24-year-olds, 1990 and 2017. Rate (per 100,000) and annualised rate of change (%), GBD 2017.

Female

		1990		2017		
Age	Rank	Cause	Rate	Cause	Rate	Rate of change (%)
10 to 14		All Cause	101.2 (90.9-110.5)	All Cause	45 (40.6-49.7)	-2.1%
	1	Typhoid fever	16.9 (8.8-29.2)	Typhoid fever	5.7 (3.0-9.6)	-2.5%
	2	Measles	9.2 (3.2-20.7)	Drowning	3.0 (1.4-4.4)	-2.1%
	3	Drowning	6.6 (2.9-10.0)	Congenital heart anomalies	2.2 (1.4-3.1)	-1.9%
	4	Lower respiratory infections	5.5 (3.1-8.5)	Lower respiratory infections	2.1 (1.3-3.2)	-2.4%
	5	Drug-susceptible tuberculosis	5.4 (3.0-8.2)	Pedestrian road injuries	2.0 (1.1-3.2)	-2.3%
	6	Pedestrian road injuries	4.8 (2.6-7.8)	Collective violence	1.8 (1.8-1.8)	401.7%
	7	Other unspecified infectious diseases	3.8 (1.8-7.1)	Malaria	1.5 (0.2-5.7)	-1.3%
	8	Congenital heart anomalies	3.6 (2.0-5.8)	Other unspecified infectious diseases	1.3 (0.7-2.2)	-2.5%
	9	Malaria	2.5 (0.2-11.6)	Other leukemia	1.3 (0.6-2.4)	-1.9%
	10	Other leukemia	2.4 (1.0-5.2)	Brain and nervous system cancer	1.2 (0.6-1.9)	-0.9%
15 to 19		All Cause	163.8 (142.8-181.0)	All Cause	74.1 (63.3-83.3)	-2.2%
	1	Drug-susceptible tuberculosis	21.2 (12.9-30.6)	Pedestrian road injuries	4.6 (2.1-8.7)	-2.4%
	2	Typhoid fever	13.0 (5.9-24.6)	Collective violence	4.5 (4.5-4.5)	739.7%
	3	Pedestrian road injuries	12.2 (5.8-21.6)	Typhoid fever	4.5 (2.1-8.5)	-2.5%
	4	Malaria	9.2 (2.2-25.6)	Malaria	3.7 (1.2-7.7)	-2.2%
	5	Maternal hemorrhage	8.7 (4.4-13.8)	Motor vehicle road injuries	3.6 (1.6-6.3)	-2.0%
	6	Motor vehicle road injuries	7.3 (2.8-14.3)	Drug-susceptible tuberculosis	2.6 (1.8-3.7)	-3.3%
	7	Lower respiratory infections	5.7 (3.5-8.9)	HIV/AIDS resulting in other diseases	2.2 (0.9-4.4)	891.3%
	8	Measles	4.9 (1.6-11.7)	Motorcyclist road injuries	2.2 (0.8-4.3)	-1.6%
	9	Motorcyclist road injuries	3.8 (0.9-8.2)	Lower respiratory infections	2.2 (1.4-3.3)	-2.4%
	10	Drowning	3.7 (2.1-5.8)	Maternal hemorrhage	2.1 (0.7-4.0)	-2.8%
20 to 24		All Cause	250.4 (212.3-277.6)	All Cause	94.7 (80.2-108.2)	-2.5%
	1	Drug-susceptible tuberculosis	46.7 (28.9-65.1)	Maternal hemorrhage	7.1 (3.0-12.6)	-2.6%
	2	Maternal hemorrhage	24.5 (14.6-36.5)	Collective violence	5.3 (5.3-5.3)	783.0%
	3	Pedestrian road injuries	15.1 (7.1-27.4)	Drug-susceptible tuberculosis	4.7 (3.0-6.8)	-3.4%
	4	Typhoid fever	9.5 (4.5-18.5)	Pedestrian road injuries	4.1 (1.8-7.7)	-2.8%
	5	Motor vehicle road injuries	9.2 (3.0-17.7)	Motor vehicle road injuries	3.7 (1.7-6.3)	-2.4%
	6	Malaria	8.2 (2.3-20.1)	Typhoid fever	3.4 (1.5-6.4)	-2.5%
	7	Lower respiratory infections	7.6 (4.6-12.3)	HIV/AIDS resulting in other diseases	3.0 (1.4-7.0)	267.8%
	8	Chronic kidney disease, unspecified cause	6.7 (3.8-10.6)	Malaria	2.8 (1.1-5.8)	-2.5%
	9	Asthma	6.6 (2.7-10.6)	Chronic kidney disease, unspecified cause	2.4 (1.4-3.8)	-2.4%
	10	Self-harm by other means	5.1 (3.1-8.5)	Lower respiratory infections	2.2 (1.4-3.5)	-2.8%

Male

		1990		2017		
Age	Rank	Cause	Rate	Cause	Rate	Rate of change (%)
10 to 14		All Cause	143.6 (126.3-167.6)	All Cause	68.2 (58.1-80.3)	-2.0%
	1	Typhoid fever	16.3 (8.2-28.9)	Drowning	7.0 (4.6-10.0)	-2.1%
	2	Drowning	15.3 (10.1-21.8)	Typhoid fever	5.7 (2.9-10.4)	-2.3%
	3	Pedestrian road injuries	11.4 (6.6-18.0)	Pedestrian road injuries	4.6 (2.5-7.4)	-2.4%
	4	Lower respiratory infections	9.4 (5.2-14.6)	Lower respiratory infections	4.2 (2.4-6.3)	-2.1%
	5	Drug-susceptible tuberculosis	7.7 (4.5-12.0)	Collective violence	3.2 (3.2-3.2)	531.0%
	6	Rabies	7.6 (1.4-22.5)	Malaria	2.8 (0.3-8.9)	-1.4%
	7	Measles	6.6 (2.3-14.8)	Congenital heart anomalies	2.3 (1.4-3.5)	-1.6%
	8	Malaria	5.0 (0.3-20.0)	Falls	2.2 (1.2-3.5)	-1.2%
	9	Asthma	4.8 (2.8-7.5)	Chronic liver disease, other causes	1.8 (1.1-3.0)	-0.8%
	10	Motor vehicle road injuries	3.8 (1.2-7.3)	Motor vehicle road injuries	1.8 (0.7-3.2)	-2.1%
15 to 19		All Cause	249.4 (212.9-309)	All Cause	153.8 (130.8-190.6)	-1.6%
	1	Motor vehicle road injuries	27.6 (12.5-45.9)	Collective violence	25.9 (25.9-25.9)	1,302.4%
	2	Pedestrian road injuries	22.7 (10.1-43.2)	Motorcyclist road injuries	13.0 (6.9-21.0)	-0.2%
	3	Malaria	18.3 (5.2-44.3)	Motor vehicle road injuries	12.2 (6.6-20.5)	-2.2%
	4	Drug-susceptible tuberculosis	15.5 (9.1-24.9)	Malaria	9.8 (3.5-19.8)	-1.7%
	5	Drowning	15.5 (9.6-23.5)	Pedestrian road injuries	8.1 (3.0-16.0)	-2.5%
	6	Motorcyclist road injuries	13.7 (5.6-26.7)	Drowning	7.9 (5.0-12.4)	-1.9%
	7	Typhoid fever	11.0 (5.0-21.1)	Self-harm by other means	5.2 (3.3-8.1)	-1.3%
	8	Self-harm by other means	8.0 (4.3-13.8)	Typhoid fever	4.2 (1.9-8.2)	-2.3%
	9	Lower respiratory infections	7.9 (4.2-12.5)	Falls	4.1 (2.6-6.3)	-0.9%
	10	Other unspecified infectious diseas	7.1 (2.8-13.4)	Lower respiratory infections	4.0 (2.3-6.4)	-1.9%
20 to 24		All Cause	379.2 (322.4-468.6)	All Cause	235.9 (195.7-291.5)	-1.7%
	1	Drug-susceptible tuberculosis	46.4 (28.2-68.7)	Collective violence	42.8 (42.8-42.9)	840.8%
	2	Motor vehicle road injuries	42.8 (20.2-68.1)	Motor vehicle road injuries	19.7 (11.6-30.7)	-2.1%
	3	Pedestrian road injuries	27.4 (12.3-54.2)	Motorcyclist road injuries	19.1 (11.7-30.0)	-0.2%
	4	Motorcyclist road injuries	20.4 (9.8-38.9)	Self-harm by other means	10.7 (6.5-16.1)	-1.2%
	5	Malaria	16.4 (5.3-43.1)	Pedestrian road injuries	10.7 (4.1-21.4)	-2.4%
	6	Self-harm by other means	15.6 (8.6-27.0)	Malaria	9.7 (3.9-21.2)	-1.4%
	7	Drowning	14.6 (8.8-22.9)	Drowning	7.6 (4.7-11.7)	-1.8%
	8	Lower respiratory infections	13.4 (7.6-22.8)	Lower respiratory infections	7.0 (3.9-11.3)	-1.8%
	9	Asthma	11.2 (6.0-17.8)	Drug-susceptible tuberculosis	7.0 (4.4-10.7)	-3.2%
	10	Chronic liver disease due to hep B	8.7 (4.6-14.0)	Falls	6.5 (4.1-10.1)	-0.8%

Legend
HIV
Sexual & Reproductive Health
Communicable Diseases
Nutrition
Assault
Unintentional Injury
Alcohol & other Drugs
Mental Disorder
Non-communicable Diseases

Table A4. Top ten causes of Years Lost to Disability in 10-24-year-olds, 1990 and 2017. Rate (per 100,000) and annualised rate of change (%), GBD 2017.

Female

Age	Rank	1990 Cause	1990 Rate	2017 Cause	2017 Rate	Rate of change (%)
10 to 14		All cause	6,783.7 (4,642.5-9,754.7)	All cause	5,203.6 (3,680.5-7,248.0)	-0.9%
	1	Dietary iron deficiency	941.6 (338.2-1,973.2)	Dietary iron deficiency	410.9 (108.8-1,111.3)	-2.3%
	2	Vitamin A deficiency	450.2 (154.5-976.5)	Migraine	410.3 (248.2-627.4)	0.1%
	3	Migraine	406.6 (243.4-623.5)	Conduct disorder	370.7 (198.7-640.0)	0.0%
	4	Conduct disorder	367.9 (203.0-629.6)	Anxiety disorders	349.0 (232.8-491.0)	0.0%
	5	Anxiety disorders	344.8 (225.6-487.7)	Atopic dermatitis	228.6 (118.5-402.4)	0.0%
	6	Atopic dermatitis	226.5 (114.2-393.3)	Low back pain	200.7 (119.3-308.7)	0.1%
	7	Ascariasis	203.4 (112.0-343.8)	Scabies	158.0 (78.3-278.7)	-0.2%
	8	Low back pain	198.3 (118.2-297.9)	Vitamin A deficiency	156.1 (41.6-407.1)	-2.5%
	9	Trichuriasis	183.1 (93.5-325.5)	Age-related/ other hearing loss	155.3 (100.9-231.7)	-0.0%
	10	Refraction disorders	182.8 (112.1-284.4)	Asthma	150.5 (89.3-231.3)	-0.1%
15 to 19		All cause	7,563.2 (5,527.9-9,996.2)	All cause	6,757.5 (4,980.2-9,122.0)	-0.4%
	1	Dietary iron deficiency	803.2 (373.6-1,392.0)	Migraine	673.8 (407.2-1,037.7)	0.0%
	2	Migraine	665.9 (402.7-1,020.2)	Dietary iron deficiency	510.2 (148.0-1,199.7)	-1.7%
	3	Anxiety disorders	417.7 (281.2-580.9)	Anxiety disorders	418.4 (276.4-579.5)	0.0%
	4	Low back pain	301.3 (180.2-460.4)	Low back pain	303.1 (181.7-471.7)	0.0%
	5	Conduct disorder	236.1 (129.5-396.3)	Conduct disorder	237.7 (126.6-399.8)	0.0%
	6	Scabies	205.2 (104.3-348.2)	Age-related/ other hearing loss	194.6 (128.8-285.3)	-0.0%
	7	Other musculoskeletal	201.9 (105.2-322.9)	Major depressive disorder	193.9 (110.6-310.4)	-0.1%
	8	Age-related/ other hearing loss	196.2 (128.7-280.8)	Scabies	192.9 (97.5-333.8)	-0.2%
	9	Major depressive disorder	194.6 (112.8-307.7)	Other musculoskeletal	177.9 (97.3-295.5)	-0.5%
	10	Refraction disorders	177.7 (111.8-269.1)	Exposure to forces of nature	162.1 (99.8-256.9)	30,226.7%
20 to 24		All cause	9,449.4 (6,852.6-12,581.3)	All cause	8,067.7 (5,821.3-10,824.5)	-0.6%
	1	Dietary iron deficiency	1,408.5 (702.9-2,423.9)	Migraine	823.3 (503.8-1,264.9)	0.1%
	2	Migraine	814.2 (482.3-1,267.4)	Dietary iron deficiency	714.0 (239.1-1,514.4)	-2.1%
	3	Other musculoskeletal	407.7 (251.3-604.1)	Anxiety disorders	409.7 (272.8-573.7)	0.0%
	4	Anxiety disorders	407.0 (276.0-563.4)	Low back pain	381.0 (232.4-596.0)	0.1%
	5	Low back pain	377.1 (230.6-583.9)	Other musculoskeletal	359.4 (213.7-535.9)	-0.4%
	6	Age-related and other hearing loss	241.3 (158.0-350.1)	Age-related/ other hearing loss	241.9 (157.1-361.2)	0.0%
	7	Diabetes mellitus type 2	225.2 (136.1-345.7)	Exposure to forces of nature	204.9 (124.5-317.0)	29,166.9%
	8	Premenstrual syndrome	215.1 (133.9-322.4)	Premenstrual syndrome	202.4 (125.5-311.2)	-0.2%
	9	Scabies	214.3 (107.8-376.2)	Scabies	201.3 (102.7-346.2)	-0.2%
	10	Chronic obstructive pulmonary disease	181.5 (124.3-247.5)	Diabetes mellitus type 2	173.2 (99.1-266.2)	-0.8%

Male

Age	Rank	1990 Cause	1990 Rate	2017 Cause	2017 Rate	Rate of change (%)
10 to 14		All cause	6,386.3 (4,388-8,767.9)	All cause	5,233.1 (3,698.5-7,475.9)	-0.7%
	1	Conduct disorder	547.6 (317.9-895.6)	Conduct disorder	553.2 (320.0-924.5)	0.0%
	2	Dietary iron deficiency	374.9 (71.3-941.9)	Migraine	373.6 (219.0-575.3)	0.1%
	3	Migraine	369.3 (219.6-562.5)	Dietary iron deficiency	309.7 (17.6-1,389.1)	-0.6%
	4	Vitamin A deficiency	350.5 (63.7-910.5)	Anxiety disorders	229.8 (148.4-337.8)	0.0%
	5	Lymphatic filariasis	334.6 (137.2-668.7)	Other congenital birth defects	229.7 (144.3-334.3)	-0.5%
	6	Other congenital birth defects	265.3 (164.0-386.2)	Age-related/ other hearing loss	206.9 (132.2-300.2)	-0.1%
	7	Anxiety disorders	227.1 (143.8-335.4)	Low back pain	181.6 (104.1-278.1)	0.1%
	8	Age-related/ other hearing loss	214.2 (135.9-310.3)	Atopic dermatitis	181.4 (90.0-322.0)	0.0%
	9	Ascariasis	201.0 (110.4-336.4)	Asthma	174.4 (101.7-270.7)	-0.4%
	10	Asthma	191.2 (112.0-295.2)	Scabies	164.1 (80.5-292.1)	-0.2%
15 to 19		All cause	7,287.1 (5,248-9,873.4)	All cause	6,421.4 (4,727.3-8,384.6)	-0.4%
	1	Migraine	603.2 (358.5-938.0)	Migraine	607.6 (352.6-934.7)	0.0%
	2	Conduct disorder	434.0 (240.9-731.9)	Conduct disorder	436.3 (245.1-729.4)	0.0%
	3	Dietary iron deficiency	410.4 (49.3-1,339.4)	Low back pain	317.3 (186.1-490.2)	0.1%
	4	Lymphatic filariasis	395.7 (170.6-785.9)	Anxiety disorders	262.3 (172.9-369.6)	0.0%
	5	Low back pain	311.2 (185.1-481.2)	Age-related/ other hearing loss	252.8 (167.8-365.1)	-0.1%
	6	Other congenital birth defects	264.3 (163.0-400.0)	Exposure to forces of nature	229.4 (140.5-353.4)	30,029.7%
	7	Age-related/ other hearing loss	260.9 (172.6-373.9)	Other congenital birth defects	226.7 (143.0-336.5)	-0.6%
	8	Anxiety disorders	259.0 (168.1-369.1)	Scabies	190.5 (98.0-326.1)	-0.2%
	9	Scabies	201.9 (101.7-352.5)	Major depressive disorder	189.0 (107.2-300.6)	0.1%
	10	Major depressive disorder	184.3 (105.6-288.1)	Bipolar disorder	150.0 (82.8-248.1)	0.0%
20 to 24		All cause	8,295.3 (6,044.8-11,007.4)	All cause	7,406.5 (5,491-9,649)	-0.4%
	1	Migraine	721.3 (433.7-1,104.8)	Migraine	730.4 (441.7-1,121.2)	0.0%
	2	Lymphatic filariasis	483.1 (201.5-946.0)	Low back pain	419.8 (248.4-652.3)	0.1%
	3	Low back pain	412.1 (250.8-629.6)	Exposure to forces of nature	362.7 (226.4-543.3)	28,541.2%
	4	Dietary iron deficiency	349.1 (58.2-991.8)	Age-related/ other hearing loss	300.1 (197.1-440.4)	-0.1%
	5	Age-related/ other hearing loss	308.5 (205.8-449.2)	Anxiety disorders	243.0 (155.4-344.2)	0.0%
	6	Other congenital birth defects	263.0 (164.9-384.8)	Other congenital birth defects	225.0 (137.5-332.5)	-0.5%
	7	Other musculoskeletal	248.2 (142.3-394.2)	Other musculoskeletal	221.7 (118.6-355.1)	-0.5%
	8	Anxiety disorders	241.1 (162.3-342.1)	Opioid use disorders	219.0 (123.3-334.6)	0.1%
	9	Opioid use disorders	210.1 (122.5-326.6)	Scabies	191.7 (98.0-333.1)	-0.3%
	10	Scabies	206.9 (105.0-359.8)	Other mental disorders	188.6 (119.6-278.2)	0.0%

Legend
HIV
Sexual & Reproductive Health
Communicable Diseases
Nutrition
Assault
Unintentional Injury
Alcohol & other Drugs
Mental Disorder
Non-communicable Diseases

Figure A2. Causes of DALYs by sex, in 10-14-year-olds. GBD 2017

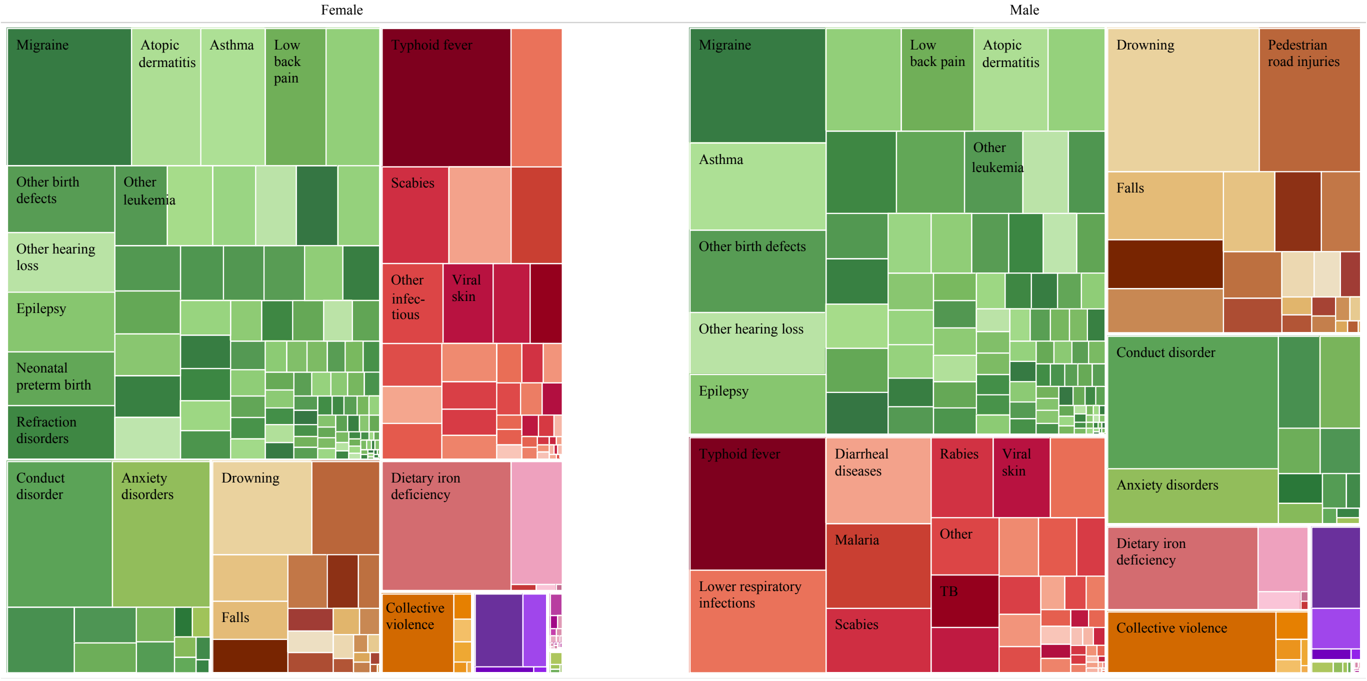

Figure A3. Causes of DALYs by sex, in 15-19-year-olds. GBD 2017

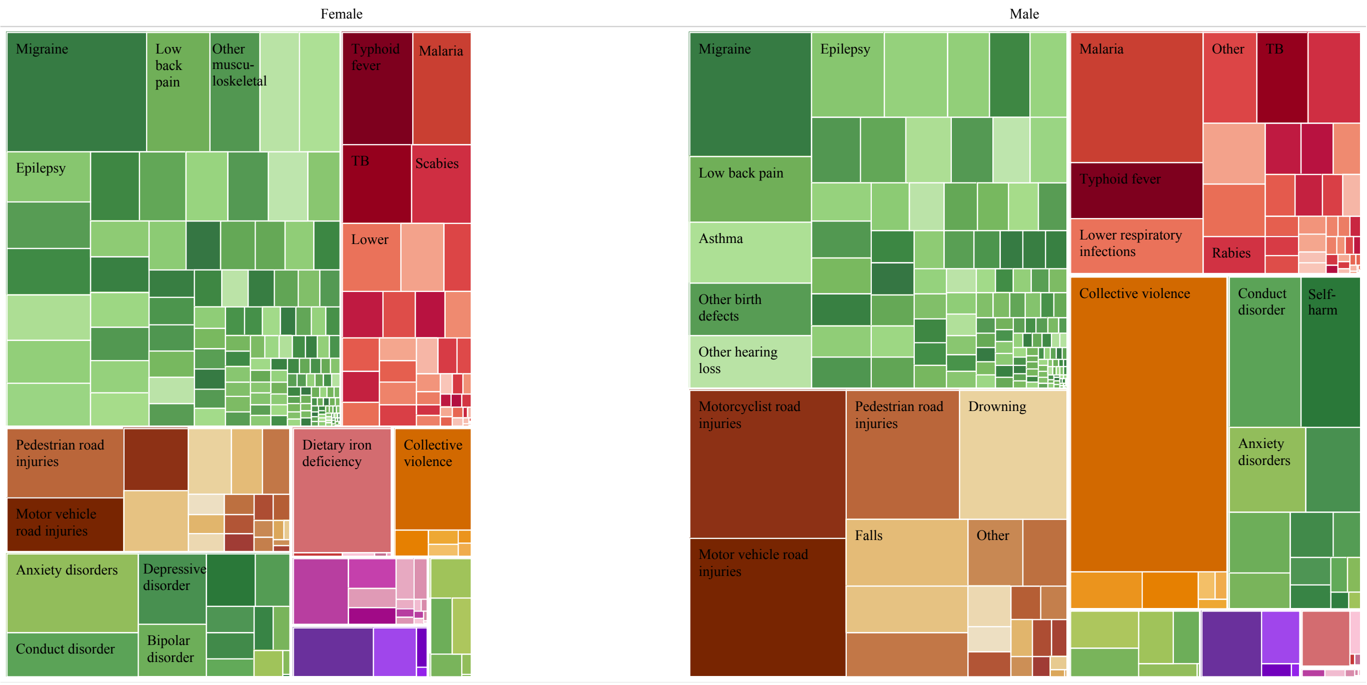

Figure A4. Causes of DALYs by sex, in 20-24-year-olds. GBD 2017

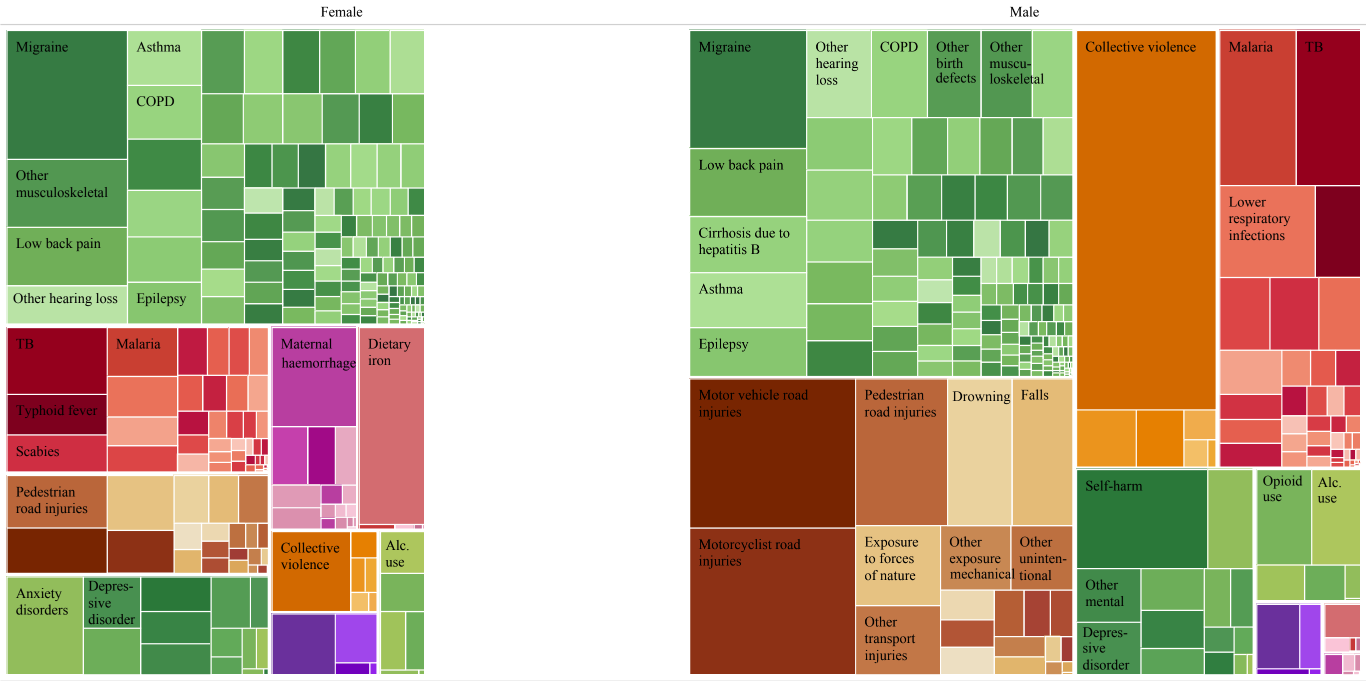

Table A5. Myanmar estimates and uncertainty intervals (where available) for all risks and social determinants, disaggregated by age and sex.

Source	Indicator	Age	Female	Male
Health risks				
GBD Study (2017)	Anaemia (total all cause) prevalence (%)	10 to 14	21.4 (7.6-48.1)	14.1 (1.3-57.7)
		15 to 19	30.6 (12.2-55.6)	16.5 (1.5-54.3)
		20 to 24	37.9 (18.5-61.5)	10.6 (0.7-37.4)
GBD Study (2016)	Binge drinking (>48g females, >60g males) past 12m, 10-24y (%)	10 to 14	0.8 (0.3-1.4)	3.9 (2.0-6.6)
		15 to 19	1.1 (0.5-2.0)	9.1 (4.5-16.1)
		20 to 24	1.3 (0.6-2.5)	14.2 (6.5-25.0)
GBD Study (2017)	Prevalence of tobacco smoking (%)	10 to 14	0.1 (0.0-0.4)	0.3 (0.1-0.7)
		15 to 19	0.4 (0.0-1.3)	6.3 (2.9-11.8)
		20 to 24	1.1 (0.2-3.6)	23.3 (12.9-38.3)
GBD Study (2017)	DALYs (rate per 100,000) due to illicit drug use	10 to 24	336.9 (228.4-482)	659 (467.6-879.6)
		15 to 19	199.1 (122-314.1)	304 (200.6-451)
		20 to 24	3.6 (2-6.2)	6.3 (3.5-10.1)
WHO Global Health Observatory (2019)	Overweight and Obesity Prevalence BMI ≥+1SD (%)	10 to 19	9.3 (5.2-14.3)	12.1 (6.2-19.9)
	Thinness Prevalence BMI ≤+2SD (%)	10 to 19	9.9 (4.7-17.2)	15.8 (7.3-26.8)
Determinants				
Myanmar DHS (2016)	Live births per 1,000 females	10 to 14	1.0	-
		15 to 19	36.0	-
		20 to 24	112.0	-
GBD Study (2017)	Live births per 1,000 females	10 to 14	0.3 (0.1-0.5)	-
		15 to 19	24.8 (20.9-29.8)	-
		20 to 24	84.7 (70.9-102.3)	-
UNESCO Institute for Statistics (2019)	Literacy (%)	15 to 24	84	85
GBD Study (2017)	Mean years of education	10 to 14	7.3 (6.7-8.0)	7.3 (6.7-8.0)
		15 to 19	8.7 (8.1-9.2)	8.3 (7.7-8.9)
		20 to 24	9.8 (9.0-10.7)	9.3 (8.4-10.2)
GBD Study (2016)	Demand for modern contraception satisfied females (%)	15 to 24	79.3 (74.4-83.7)	-
International Labour Organization (2019)	Youth not in education, employment or training (%)	15 to 24	23.6	10.6

Myanmar DHS (2016)	Child Marriage - Adolescents married before age 15 (%)<br>- Adolescents married before age 18 (%)	20 to 24	2<br>16	0<br>5
Myanmar DHS (2016)	Educational Attainment (%) - none<br>- some primary<br>- completed primary<br>- some secondary<br>- completed secondary<br>- higher	20 to 24	7.5<br>15.6<br>15.9<br>41.9<br>6.1<br>13	7.3<br>14.6<br>12<br>49.3<br>7.1<br>9.7

Figure A5. Full Legend for Figure 2.

- Communicable, Acute hepatitis A
- Communicable, Acute hepatitis B
- Communicable, Acute hepatitis C
- Communicable, Acute hepatitis E
- Communicable, African trypanosomiasis
- Communicable, Ascariasis
- Communicable, Cellulitis
- Communicable, Chagas disease
- Communicable, Cutaneous and mucocutaneous leis..
- Communicable, Cystic echinococcosis
- Communicable, Cysticercosis
- Communicable, Dengue
- Communicable, Diarrheal diseases
- Communicable, Diphtheria
- Communicable, Ebola
- Communicable, Encephalitis
- Communicable, Extensively drug-resistant tubercul..
- Communicable, Food-borne trematodiases
- Communicable, Fungal skin diseases
- Communicable, Guinea worm disease
- Communicable, H influenzae type B meningitis
- Communicable, Hookworm disease
- Communicable, Invasive Non-typhoidal Salmonella..
- Communicable, Latent tuberculosis infection
- Communicable, Leprosy
- Communicable, Lower respiratory infections
- Communicable, Lymphatic filariasis
- Communicable, Malaria
- Communicable, Measles
- Communicable, Meningococcal meningitis
- Communicable, Neonatal sepsis and other neonatal i..
- Communicable, Onchocerciasis
- Communicable, Other infectious
- Communicable, Other intestinal infectious diseases
- Communicable, Other meningitis
- Communicable, Other neglected tropical
- Communicable, Otitis media
- Communicable, Paratyphoid fever
- Communicable, Pneumococcal meningitis
- Communicable, Pyoderma
- Communicable, Rabies
- Communicable, Scabies
- Communicable, Schistosomiasis
- Communicable, TB Drug-susceptible
- Communicable, TB Multidrug-resistant without ext..
- Communicable, Tetanus
- Communicable, Trachoma
- Communicable, Trichuriasis
- Communicable, Typhoid fever
- Communicable, Upper respiratory infections
- Communicable, Varicella and herpes zoster
- Communicable, Viral skin diseases
- Communicable, Visceral leishmaniasis
- Communicable, Whooping cough
- Communicable, Yellow fever
- Communicable, Zika virus
- HIV, HIV/AIDS - Drug-susceptible Tuberculosis
- HIV, HIV/AIDS - Extensively drug-resistant Tuber..
- HIV, HIV/AIDS - Multidrug-resistant Tuberculosis ..
- HIV, HIV/AIDS resulting in other diseases
- Inj_assault, Collective violence
- Inj_assault, Conflict and terrorism
- Inj_assault, Physical violence by firearm
- Inj_assault, Physical violence by other means
- Inj_assault, Physical violence by sharp object
- Inj_assault, Sexual violence
- Inj_unintentional, Adverse effects of medical treatm..
- Inj_unintentional, Cyclist road injuries
- Inj_unintentional, Drowning
- Inj_unintentional, Environmental heat and cold exp..
- Inj_unintentional, Exposure to forces of nature
- Inj_unintentional, Falls
- Inj_unintentional, Fire, heat, and hot substances
- Inj_unintentional, Foreign body in eyes
- Inj_unintentional, Foreign body in other body part
- Inj_unintentional, Motor vehicle road injuries
- Inj_unintentional, Motorcyclist road injuries
- Inj_unintentional, Non-venomous animal contact
- Inj_unintentional, Other exposure to mechanical for..
- Inj_unintentional, Other road injuries
- Inj_unintentional, Other transport injuries
- Inj_unintentional, Other unintentional injuries
- Inj_unintentional, Pedestrian road injuries
- Inj_unintentional, Poisoning by carbon monoxide
- Inj_unintentional, Poisoning by other means
- Inj_unintentional, Pulmonary aspiration and foreign..
- Inj_unintentional, Unintentional firearm injuries
- Inj_unintentional, Venomous animal contact
- NCD, Acne vulgaris
- NCD, Acute glomerulonephritis
- NCD, Acute lymphoid leukemia
- NCD, Acute myeloid leukemia
- NCD, Age-related macular degeneration
- NCD, Alcoholic cardiomyopathy
- NCD, Alopecia areata
- NCD, Alzheimer's
- NCD, Aortic aneurysm
- NCD, Appendicitis
- NCD, Asbestosis
- NCD, Asthma
- NCD, Atopic dermatitis
- NCD, Atrial fibrillation and flutter
- NCD, Benign and in situ cervical and uterine neopla..
- NCD, Benign and in situ intestinal neoplasms
- NCD, Benign prostatic hyperplasia
- NCD, Bladder cancer
- NCD, Brain and nervous system cancer
- NCD, Breast cancer
- NCD, Caries of deciduous teeth
- NCD, Caries of permanent teeth
- NCD, Cataract
- NCD, Cervical cancer
- NCD, Chronic lymphoid leukemia
- NCD, Chronic myeloid leukemia
- NCD, Cirrhosis due to alcohol use
- NCD, Cirrhosis due to hepatitis B
- NCD, Cirrhosis due to hepatitis C
- NCD, Cirrhosis due to NASH
- NCD, Cirrhosis due to other causes
- NCD, CKD due to DMT1
- NCD, CKD due to DMT2
- NCD, CKD due to glomerulonephritis
- NCD, CKD due to hypertension
- NCD, CKD due to other causes
- NCD, Coal workers pneumoconiosis
- NCD, Colon and rectum cancer
- NCD, Congenital heart
- NCD, Congenital musculoskeletal
- NCD, Contact dermatitis
- NCD, COPD
- NCD, Decubitus ulcer
- NCD, Diabetes T1
- NCD, Diabetes T2
- NCD, Digestive congenital anomalies
- NCD, Down syndrome
- NCD, Edentulism and severe tooth loss
- NCD, Endocarditis
- NCD, Endocrine, metabolic, blood, and immune dis..
- NCD, Endometriosis
- NCD, Epilepsy
- NCD, Esophageal cancer
- NCD, Female infertility
- NCD, G6PD deficiency
- NCD, G6PD trait
- NCD, Gallbladder and biliary diseases
- NCD, Gallbladder and biliary tract cancer
- NCD, Gastritis and duodenitis
- NCD, Gastroesophageal reflux disease
- NCD, Genital prolapse
- NCD, Glaucoma
- NCD, Gout
- NCD, Hemolytic disease and other neonatal jaundice
- NCD, Hodgkin lymphoma
- NCD, Hypertensive heart disease
- NCD, Inflammatory bowel disease
- NCD, Inguinal, femoral, and abdominal hernia
- NCD, Interstitial lung disease and pulmonary sarcoi..
- NCD, Intracerebral hemorrhage
- NCD, Ischemic heart disease
- NCD, Ischemic stroke
- NCD, Kidney cancer
- NCD, Klinefelter syndrome
- NCD, Larynx cancer
- NCD, Lip and oral cavity cancer
- NCD, Liver cancer due to alcohol use
- NCD, Liver cancer due to hepatitis B
- NCD, Liver cancer due to hepatitis C
- NCD, Liver cancer due to NASH
- NCD, Liver cancer due to other causes
- NCD, Low back pain
- NCD, Male infertility
- NCD, Malignant skin melanoma
- NCD, Mesothelioma
- NCD, Migraine
- NCD, Motor neuron disease
- NCD, Multiple myeloma
- NCD, Multiple sclerosis
- NCD, Myelodysplastic, myeloproliferative, and oth..
- NCD, Myocarditis
- NCD, Nasopharynx cancer
- NCD, Near vision loss
- NCD, Neck pain
- NCD, Neonatal encephalopathy due to birth asphyxi..
- NCD, Neonatal preterm birth
- NCD, Neural tube defects
- NCD, Non-Hodgkin lymphoma
- NCD, Non-melanoma skin cancer (basal-cell carcin..
- NCD, Non-melanoma skin cancer (squamous-cell c..
- NCD, Non-rheumatic calcific aortic valve disease
- NCD, Non-rheumatic degenerative mitral valve dise..
- NCD, Orofacial clefts
- NCD, Osteoarthritis
- NCD, Other benign and in situ neoplasms
- NCD, Other birth defects
- NCD, Other cardiomyopathy
- NCD, Other cardiovascular
- NCD, Other chromosomal abnormalities
- NCD, Other chronic respiratory
- NCD, Other digestive
- NCD, Other gynecological
- NCD, Other hearing loss
- NCD, Other hemoglobinopathies
- NCD, Other leukemia
- NCD, Other malignant neoplasms
- NCD, Other musculoskeletal
- NCD, Other neonatal disorders
- NCD, Other neurological
- NCD, Other non-rheumatic valve diseases
- NCD, Other oral disorders
- NCD, Other pharynx cancer
- NCD, Other pneumoconiosis
- NCD, Other sense organ
- NCD, Other skin
- NCD, Other urinary diseases
- NCD, Other vision loss
- NCD, Ovarian cancer
- NCD, Pancreatic cancer
- NCD, Pancreatitis
- NCD, Paralytic ileus and intestinal obstruction
- NCD, Parkinson's disease
- NCD, Peptic ulcer disease
- NCD, Periodontal diseases
- NCD, Peripheral artery disease
- NCD, Polycystic ovarian syndrome
- NCD, Premenstrual syndrome
- NCD, Prostate cancer
- NCD, Pruritus
- NCD, Psoriasis
- NCD, Refraction disorders
- NCD, Rheumatic heart disease
- NCD, Rheumatoid arthritis
- NCD, Seborrhoeic dermatitis
- NCD, Sickle cell disorders
- NCD, Sickle cell trait
- NCD, Silicosis
- NCD, Stomach cancer
- NCD, Subarachnoid hemorrhage
- NCD, Sudden infant death syndrome
- NCD, Tension-type headache
- NCD, Testicular cancer
- NCD, Thalassemias
- NCD, Thalassemias trait
- NCD, Thyroid cancer
- NCD, Tracheal, bronchus, and lung cancer
- NCD, Turner syndrome
- NCD, Urinary tract infections
- NCD, Urogenital congenital anomalies
- NCD, Urolithiasis
- NCD, Urticaria
- NCD, Uterine cancer
- NCD, Uterine fibroids
- NCD, Vascular intestinal disorders
- NCD_AOD, Alcohol use
- NCD_AOD, Amphetamine use
- NCD_AOD, Cannabis use disorders
- NCD_AOD, Cocaine use disorders
- NCD_AOD, Opioid use
- NCD_AOD, Other drug use
- NCD_MH, Anorexia nervosa
- NCD_MH, Anxiety disorders
- NCD_MH, Attention-deficit/hyperactivity disorder
- NCD_MH, Autism spectrum disorders
- NCD_MH, Bipolar disorder
- NCD_MH, Bulimia nervosa
- NCD_MH, Conduct disorder
- NCD_MH, Depressive disorder
- NCD_MH, Dysthymia
- NCD_MH, Idiopathic developmental intellectual dis
- NCD_MH, Other mental disorder
- NCD_MH, Schizophrenia
- NCD_MH, Self-harm
- NCD_MH, Self-harm by firearm
- Nutrition, Dietary iron deficiency
- Nutrition, Iodine deficiency
- Nutrition, Other nutritional deficiencies
- Nutrition, Protein-energy malnutrition
- Nutrition, Vitamin A deficiency
- SRH, Chlamydial infection
- SRH, Ectopic pregnancy
- SRH, Genital herpes
- SRH, Gonococcal infection
- SRH, Indirect maternal deaths
- SRH, Late maternal deaths
- SRH, Maternal abortion and miscarriage
- SRH, Maternal deaths HIV/AIDS
- SRH, Maternal hemorrhage
- SRH, Maternal hypertensive disorders
- SRH, Maternal obstructed labor and uterine rupture
- SRH, Maternal sepsis
- SRH, Other maternal disorders
- SRH, Other STI
- SRH, Syphilis
- SRH, Trichomoniasis
